# A Global Meta‐Analysis of Water Use Efficiency Proxies Reveals That UV Radiation Decreases Transpiration Without Improving WUE

**DOI:** 10.1111/pce.15643

**Published:** 2025-05-28

**Authors:** Marcel A. K. Jansen, Alexander Ač, John Grace, Otmar Urban

**Affiliations:** ^1^ Global Change Research Institute of the Czech Academy of Sciences Brno Czech Republic; ^2^ School of Biological, Earth and Environmental Sciences, Environmental Research Institute University College Cork Cork Ireland; ^3^ School of GeoSciences University of Edinburgh Edinburgh UK

**Keywords:** photosynthesis, stable carbon isotopes, stomata, ultraviolet radiation

## Abstract

Plant water use efficiency (WUE) links physiological processes to ecosystem‐scale carbon and water cycles, making it a crucial parameter for climate change adaptation modelling. Climate and stratospheric ozone dynamics expose plants to varying intensity of ultraviolet‐B radiation (UV‐B), which affects stomatal function and transpiration. This meta‐analysis evaluates UV‐B effects on WUE using gas exchange and isotopic proxies. While UV‐B radiation reduces stomatal conductance and transpiration, it also suppresses photosynthesis, particularly under non‐saturating light. As a result, WUE remains unchanged or declines in UV‐B exposed plants, depending on the measurement method. Instantaneous gas exchange‐based WUE proxies indicate a decrease, whereas isotope‐based proxies, integrating long‐term fluxes, show no significant UV‐B effect. The suppression of photosynthesis due to UV‐B occurs only when UV‐B lamps are used to increase the UV‐B dose; when UV‐B is excluded under field conditions there is no significant effect on WUE. Only some field studies report improved WUE under ambient UV‐B, suggesting potential adaptive benefits. Overall, the findings challenge the assumption that UV‐B‐induced decreases in transpiration enhance WUE. Instead, they highlight a complex interplay between UV radiation, photosynthesis, and stomatal regulation, emphasizing the need to reconsider UV‐B's role in plant water relations under future climate conditions.

## 1 Introduction

The discovery of thinning of the stratospheric ozone layer in 1980s (Farman et al. 1985), and the concomitant increase in ultraviolet‐B (UV‐B) radiation in the biosphere, triggered concerns about the effects of such radiation on living organisms (Stapleton 1992; Britt 1995). However, thanks to multilateral action, that is, the Montreal Protocol and its Amendments, emissions of ozone‐depleting substances have been dramatically reduced, and there are now early signs of stratospheric ozone layer recovery (McKenzie et al. 2019).

Yet, interactive effects between climate change and stratospheric ozone pose a potential threat to the stratospheric ozone layer, and thus affect UV‐B radiation levels in the biosphere (Bornman et al. 2019; Barnes et al. 2023; Chatzopoulou et al. 2024). UV‐B radiation, while strongly scattered by Rayleigh molecular interactions in the atmosphere, is less attenuated by clouds compared to visible light, allowing significant UV‐B penetration even under overcast conditions. These wavelength‐dependent interactions underscore the diffuse nature of UV‐B radiation, complicating predictions of its variability under changing climatic drivers.

A recent UNEP‐EEAP assessment detailed an increase in the UV‐index of 3–8% over the tropics and mid‐latitudes depending on the greenhouse gas scenario and considering altered cloud cover and aerosol concentration (Barnes et al. 2023). The ongoing decline in global aerosol emissions is contributing to the increased Earth energy imbalance (Hodnebrog et al. 2024), a reduction in atmospheric albedo due to diminished low cloud formation and, as a consequence, higher UV‐B doses reaching the biosphere (Goessling et al. 2024). This has significant regional radiative forcing effects (Schumacher et al. 2024). Furthermore, climate change intervention (‘geoengineering’) involving injection of large amounts of gaseous sulphur dioxide (SO_2_) into the lower stratosphere poses a further potential future risk to stratospheric ozone (Neale et al. 2025). Thus, an issue of increasing concern is the exposure of organisms to new and rapidly changing combinations of solar UV radiation (combination of UV‐B and UV‐A wavebands in sunlight) and climate parameters such as high temperatures and low water availability (Bornman et al. 2019; Barnes et al. 2023).

Negative effects of UV‐B radiation on plants, particularly on DNA and the photosynthetic machinery, have been extensively detailed (cf. Jansen et al. 1998; Kataria et al. 2014; Podolec et al. 2021; Lingwan 2024). However, experimental evidence shows that plants acclimated to realistic doses of UV‐B radiation, under otherwise favourable growth conditions, rarely experience stress (Bornman et al. 2019). Rather, plants undergo comprehensive reprogramming of their physiology and morphology, a process mediated by the UV‐B photoreceptor UVR8 (Jenkins 2014; Jenkins 2017; Rai et al. 2020; Podolec et al. 2021) and with relevance across a wide range of natural growth conditions (Neugart et al. 2024).

These UV‐induced responses include wide ranging changes to hormonal signalling (Vanhaelewyn et al. 2016), and associated changes in gene expression, accumulation of various secondary metabolites, including flavonoids, terpenoids, alkaloids, glucosinolates, polyamines, and tocopherols (cf. Barnes et al. 2023; Neugart et al. 2024), antioxidant defences (Agarwal 2007; Hideg et al. 2013), DNA repair capacity (Britt 1995), and stomatal regulation (Nogués et al. 1999; Ač et al. 2024). UVR8 has been associated with changes in stomatal density (Wargent et al. 2009), as well as control of stomatal aperture (Ge et al. 2020). Morphological changes include the development of a more compact phenotype characterized by shorter stems, increased branching, and/or smaller but thicker leaves (Barnes et al. 1996; Robson et al. 2015). The functional importance of these physiological and morphological changes is not always well understood; while some of these changes mitigate UV‐B radiation damage or enhance repair of such damage, others are speculatively linked to increased resistance to oxidative stress, particularly drought (Bandurska et al. 2013; Aphalo and Sadras 2022).

In natural environments, high intensity of UV‐B radiation often coincides with exposure to high photosynthetically active radiation, drought, and elevated temperatures, potentially causing additive or synergistic stress effects in plants (Jansen et al. 2019). Conversely, UV‐B radiation may also induce cross‐resistance to other stressors (Jansen et al. 2019). For example, a recent meta‐analysis revealed that the combined effects of UV‐B and drought on plant stress responses—such as leaf area reduction and plant height inhibition—are less‐than‐additive, implying a degree of cross‐resistance between these stressors (Jansen et al. 2022). This phenomenon may stem from overlapping molecular, biochemical, physiological or morphological responses to UV‐B and drought, as recently reported (Bandurska et al. 2013; Jansen et al. 2022). Understanding the mechanisms underlying plant responses to co‐occurring stressors is critical in the context of rapidly changing combinations of solar UV radiation and climate factors, while also offering opportunities to develop novel plant manipulation strategies, such as priming.

A recent meta‐analysis revealed strong evidence for UV‐B‐mediated decreases in stomatal aperture and size (Ač et al. 2024). Stomatal conductance is proportional to mean stomatal pore area, as well as stomatal density (stomata per unit epidermal area), while it is inversely proportional to the average pore length—a morphological/anatomical trait sensitive to UV‐B, as shown in Ač et al. (2024). Decreased stomatal aperture can potentially reduce water loss through transpiration, but also limit CO_2_ diffusion into the leaf mesophyll, thereby reducing photosynthetic carbon assimilation. Furthermore, reduced cooling of leaves and vegetation may impair photosynthetic performance, particularly in hotter climates where photosynthesis already operates close to the optimal temperature threshold (Doughty et al. 2023; Crous et al. 2025). However, plant responses to UV‐B are multifaceted, as evidenced by the observation that UV‐B exposure can also increase stomatal density (Ač et al. 2024). This increase may partially offset the effects of stomatal closure, with significant global implications for ecosystems.

Thus, it can be hypothesized that the effects of UV‐B radiation on plant water use will be determined by the balance of potentially competing influences. The aim of this paper is to assess the net effect of UV‐B‐mediated changes in stomatal behaviour on water use efficiency (WUE) at leaf‐level; an issue directly relevant in the context of a changing climate. WUE represents the balance between carbon assimilation and water loss, and is a key indicator of the efficiency with which plants use water to produce biomass (Hatfield and Dold 2019). Parameters underlying WUE such as net photosynthesis, leaf transpiration and stomatal conductance are affected by multiple environmental factors, often with unknown dose‐responses and/or wavelength‐dependencies, and this contributes to variable, and seemingly contradictory, data published in the literature. Therefore, this study assesses responses of WUE to UV exposure through a meta‐analysis approach, interrogating a substantial number of published papers to extract global response trends.

As a crucial (eco)physiological parameter, WUE links plant‐level processes to ecosystem and global scales. Thus, understanding WUE responses to various environmental drivers is important for predicting plant/ecosystem resilience in future climate conditions. WUE is widely used in global models to link gross primary production (GPP) and evapotranspiration (ET). For example, the Community Land Model (CLM) and Dynamic Global Vegetation Models (DGVMs) simulate vegetation responses to environmental drivers or even biome shifts (Bonan and Levis 2006; Oleson et al. 2008; Eckes‐Shephard et al. 2021). Therefore, accurate parametrization and understanding the mechanisms of how WUE varies across environmental conditions, plant species, and geographical regions are essential for improving these models. WUE is not a static trait; it varies with environmental conditions, diurnal cycles, and exposure to stressors like drought. Therefore, this study also investigates both instantaneous WUE measurements and integrated seasonal estimates based on isotopic analyses to provide comprehensive insights into WUE dynamics and to contribute to the development of better WUE measurement protocols.

In this paper, three basic hypotheses are tested. Firstly, it is hypothesised that UV radiation impacts plant water use efficiency by altering physiological processes, including photosynthesis, stomatal behaviour, and transpiration, which collectively determine WUE. Here we test whether the magnitude and direction of these effects are influenced by factors such as UV dose, exposure duration, plant functional type, and experimental growth conditions.

Secondly, it is hypothesised that long‐term acclimation and natural growth environments moderate the impacts of UV radiation on WUE. This hypothesis assumes that plants have significant biochemical and morphological capacities to acclimate to long‐term UV exposure under field conditions. We expect that such acclimation reduces the sensitivity of WUE to UV radiation compared to short‐term treatments or controlled environments, where acclimation mechanisms are limited.

Thirdly, it is hypothesised that isotope‐based WUE proxies, which integrate plant responses over extended time periods, may provide a more robust metric for assessing plant acclimation to UV radiation than instantaneous gas‐exchange‐based WUE proxies.

## 2 Materials and Methods

### 2.1 Literature Search

The primary literature search was performed in the Scopus database. The following combinations of search terms were used; (1) “UV*”, “net photosynthesis” and “stomatal conductance”; (2) “UV*”, “net photosynthesis” and “transpiration” and (3) “UV*” and “WUE”. To search for WUE studies based on stable carbon isotope discrimination, the combination “UV*”, “carbon isotope*”, and “water use efficiency” was used (4). The Boolean operator AND was used to combine keywords.

The initial search with keywords (1)–(3) identified 124 papers, while application of keyword set (4) yielded an additional 17 papers (Supplementary Figure S1). After removing 19 duplicate records, 122 unique records were screened. At this screening stage, eight studies were excluded due to their research focus on either UV‐C or UV‐A radiation. As a result, the selected papers focus on studies in which plants were exposed to UV‐B or a combination of UV‐B and UV‐A radiation. A further 16 papers were excluded as they were written in a language other than English, the experimental design could not be adequately assessed and/or the paper summarized the results of previous peer‐review studies. The texts of the remaining 98 records were assessed in detail, resulting in the exclusion of further 24 papers due to reasons such as a focus on aquatic species, insufficient description of experimental conditions, UV‐B treatments that included UV‐C, or the nonavailability of a complete version of the manuscript. In total, 74 publications, published in peer‐reviewed scientific journals over the past 34 years (1990–2024; Supplementary Figure S2), met the inclusion criteria and were incorporated into the analysis presented in this paper. See PRISMA (Preferred Reporting Items for Systematic Reviews and Meta‐Analyses) flow diagram for further details (Supplementary Figure S1).

Publications detailing responses of multiple plant species, cultivars, or developmental stages, or where effects of multiple UV‐doses and/or treatment durations were split into separate ‘case studies’, resulting in a total of 201 case studies. These case studies were treated as being statistically independent.

### 2.2 Data Compilation and Processing

Papers were analysed for physiological activities pertaining to overall water use:

1. Stomatal conductance (*G*s), defined as the rate of water loss through the stomata of a leaf, per unit of surface area. Where studies presented stomatal limitation or stomatal resistance, the inverse was used for the analysis.
2. Leaf transpiration (*E*), defined as the rate of water loss through the stomata per unit of leaf surface area.
3. Net photosynthesis (*A*), which relates to either the rate of CO_2_ assimilation under ambient, non‐saturating light conditions, or CO_2_ assimilation rates under saturating light conditions (arbitrarily defined as intensities of photosynthetically active radiation > 1000 µmol m^–2^ s^–1^).
4. Water use efficiency (WUE) derived from gas‐exchange data or carbon stable isotopes.

Information was collected on plant functional types (woody plants = broadleaved and coniferous trees (Tb, Tc), and shrubs (Sh); non‐woody plants = herbs (He; non‐woody dicotyledonous plants) and grasses (Gr; non‐woody monocotyledonous plants), growth conditions (growth chamber, greenhouse or field), UV‐exposure approach (UV‐exclusion or supplementation) and UV‐exposure conditions (UV irradiance and dose, and duration of the UV‐exposure). In the analysed studies, UV treatment refers to exposure to either UV‐B alone or a combination of UV‐B and a UV‐A background (e.g., natural sunlight studies or studies using UV‐B tubes that emit some UV‐A radiation). Pure UV‐A exposure studies were excluded. The “exclusion type” experiments, in which control plants were exposed to filtered natural sunlight, were categorized as below ambient UV dose experiments (aUV). The “supplementary type” experiments were, somewhat arbitrarily, grouped into three broad categories based on the available information about the UV dose above the ambient background in kJ m^–2^ day^–1^: (1) low UV dose experiments (Sup_Low; below 5 kJ m^−2^ day^−1^), (2) medium UV dose experiments (Sup_Med; between 5 and 10 kJ m^–2^ day^–1^), and (3) high UV dose experiments (Sup_High; above 10 kJ m^–2^ day^–1^ and with a maximum of 190 kJ m^–2^ day^–1^). For context, ambient yearly average UV‐B values across the globe range between 0.4 and 8.6 kJ m^–2^ day^–1^ (source: https://www.ufz.de/gluv/index.php?en=32435). As natural UV doses vary with latitude and altitude (Barnes et al. 2023), these categories do not necessarily reflect ambient doses at a specific location.

In terms of exposure duration, studies were categorized as short (duration experiment ≤ 30 days; Dur_short), medium (30–90 days; Dur_med) and long‐term experiments ( > 90 days; Dur_long).

The data obtained were compiled in Excel together with details of the citation, Latin plant name, cultivar name, and growth conditions (Supporting Table S1). During subsequent data processing, untreated plants represented the control in UV‐supplementation studies, while UV‐shielded plants were considered the control in UV‐exclusion studies. To quantify the magnitude of net UV effect, relative changes induced by UV radiation in % were calculated as [(UV_treated_ – UV_control_)/UV_control_] × 100.

### 2.3 Standardisation of Units in the Data Set

The mean values of the gas‐exchange parameters, *A*, *G*s, and *E*, as well as the ratio of the two stable carbon isotopes (δ^13^C; ^13^C/^12^C) were compiled along with the reported measures of their variability (standard error, SE, or standard deviation, SD), and the number of replicates (*n*). The values of gas‐exchange parameters were standardised as μmol CO_2_ m^–2^ s^–1^ for *A*, mol H_2_O m^–2^ s^–1^ for *G*s, and mmol H_2_O m^–2^ s^–1^ for *E*. In case studies where only SE values were reported, these were converted to SD using the following formula:

(1) $$\mathrm{SD}=\mathrm{SE}\times \sqrt{n}$$

### 2.4 Water Use Efficiency Calculations

Two measures of water use efficiency (WUE) were compiled:

1. Gas‐exchange‐based WUE, reflecting the instantaneous physiological status of plant, calculated from the *A*, *G*s, and *E* values, and
2. Isotope‐based WUE, reflecting long‐term integrative acclimation based on δ^13^C values of plant organs accumulated over a considerable time.

Stomatal‐based (sWUE) and transpiration‐based (eWUE) water use efficiencies were defined as:

(2) $$\mathrm{sWUE}=\frac{A}{\mathrm{Gs}}$$

(3) $$\mathrm{eWUE}=\frac{A}{E}$$

For studies where sWUE or eWUE were not reported, Equations 2 and 3 were used to calculate values. The standard deviation of WUE (σ_f_) was calculated in these cases as:

(4) $$\sigma_{f}=\sqrt{{\left(\frac{\partial f}{\partial x_{1}}\right)}^{2}\sigma_{x1}^{2}+{\left(\frac{\partial f}{\partial x_{2}}\right)}^{2}\sigma_{x2}^{2}},$$

where *f* is the function (either sWUE = *A*/*G*s or eWUE = *A*/*E*), *x*1 and *x*2 are the variables (*A* and *G*s or *A* and *E*), and *σ*_*x*1_ and *σ*_*x*2_ are calculated standard deviations.

Gas‐exchange measurements were reported under both saturating and non‐saturating intensities of PAR. Accordingly, the following designations were used: sWUEsat and eWUEsat for measurements under saturating light, and sWUEnon and eWUEnon for measurements under non‐saturating light.

Isotope‐based WUE (isWUE) was calculated from the stable carbon isotope composition of plant tissues (δ^13^Cplant) determined as:

(5) $$\delta^{13}\mathrm{Cplant}=(\frac{R_{\mathrm{plant}}}{R_{\mathrm{standard}}}-1)\times 1000,$$

where *R*_plant_ and *R*_standard_ are the ^13^C/^12^C ratios in the plant tissues and the isotopic standard (Vienna PeeDee Belemnite, VPDB), respectively. The δ^13^Cplant value reflects both biochemical and diffusional processes during photosynthesis (Farquhar et al. 1989). Where only δ^13^Cplant was reported, isWUE was calculated based on the intercellular and atmospheric CO_2_ concentrations. The intercellular CO_2_ concentration (*C*i) was estimated using a simplified model that neglects post‐photosynthetic carbon isotope fractionations and temperature effects (Mathias and Hudiburg 2022):

(6) Ci=ΔC−a13b−a×Ca,

where *a*, and *b* are correction factors accounting for carbon isotope fractionation due to CO_2_ diffusion across the stomata (4.4‰), and carboxylation by Rubisco (27‰), respectively. *C*a is the atmospheric CO_2_ concentration in μmol CO_2_ mol^–1^. If not reported in the study, the global mean atmospheric CO_2_ concentration for the study year was used, following data from the National Oceanic and Atmospheric Administration (NOAA) database (https://gml.noaa.gov/ccgg/trends/gl_data.html). Carbon isotope discrimination (Δ^13^C in ‰), which accounts for the carbon isotope composition of the atmosphere, was calculated as:

(7) $$\Delta^{13}C=\frac{\delta^{13}\mathrm{Catm}-\delta^{13}\mathrm{Cplant}}{1+\frac{\delta^{13}\mathrm{Cplant}}{1000}},$$

where δ^13^Catm and δ^13^Cplant represent the stable carbon isotope ratios in the atmosphere and plant (leaf) tissue, respectively. δ^13^Catm values were retrieved from Graven et al. (2020) or from direct measurements at Mauna Loa for the corresponding study year, following NOAA data (https://gml.noaa.gov/ccgg/trends/data.html). Since all δ^13^C values were reported for leaves, corrections for the post‐photosynthetic fractionation were not applied.

Values of isWUE, expressed in μmol mol^–1^, were calculated (Farquhar et al. 1989) as:

(8) $$\mathrm{isWUE}=\frac{\left(\mathrm{Ca}-\mathrm{Ci}\right)}{1.6}.$$

The coefficient 1.6 accounts for the ratio of stomatal conductance to CO_2_ and water vapour. To calculate the standard deviation of isWUE (σ_isWUE_) based on the mean and standard deviation of δ¹³Cplant, error propagation was applied through Equations 6(6), (7), (8). The standard deviation of isWUE is:

(9) $$\sigma_{\mathrm{isWUE}}=\left|\frac{\partial \mathrm{isWUE}}{\partial \delta^{13}\mathrm{Cplant}}\right|\times \sigma_{\delta^{13}\mathrm{Cplant}}.$$

The error propagation can be approximated as:

(10) $$\sigma_{\mathrm{isWUE}}\approx \frac{\mathrm{Ca}}{1.6\times b}\times \sigma_{\delta^{13}\mathrm{Cplant}}.$$

*C*a is the atmospheric CO_2_ concentration and *b* is the fractionation factor during carboxylation by Rubisco (27‰).

### 2.5 Meta‐Analysis

The standard difference in the means (SDM), sometimes referred to as the standard means difference (SMD), was used to calculate the summary (net) effect across all case studies. SDM quantitatively evaluates the size of the intervention effect in each case study relative to the variability observed within that study. For the calculation of SDM, the standard deviation and the number of replicates (*n*) were recorded for both control and treated plants. In cases where only the sample size (*n*) and *p*‐values of treatment effect were reported, the SDM was determined using the following equation:

(11) $$\mathrm{SDM}=\;\frac{\bar{{\mathrm{UV}}_{1}}-\;\bar{{\mathrm{UV}}_{2}}}{\sqrt{\frac{\sigma_{1}^{2}\left(n_{1}-1\right)+\sigma_{2}^{2}(n_{2}-1)}{n_{1}+\;n_{2}-2\;}}},$$

where $\bar{{\mathrm{UV}}_{1}}$ and σ_1_^2^ are the mean and standard deviation of the given parameter for the UV‐treated plants, calculated from n_1_ observations, while $\bar{{\mathrm{UV}}_{2}}$ and σ_2_^2^ are the mean and standard deviation for the control plants, calculated from n_2_ observations. For further details, refer to Ač et al. (2015).

In this study, the random effect model was applied to calculate the net treatment effect. This approach utilized the Z‐value, which reflects the proximity of an individual value to the group mean. This variability is expected due to differences in plant species, experimental conditions, setup, and measurement techniques (Borenstein et al. 2009). The summary effect size and statistical significance for all considered case studies are reported as the SDM along with the associated *p*‐value. The variability and spread of the effect are expressed as the 95% confidence interval (CI). To assess statistically significant (*p* < 0.05) differences between subgroups (e.g., plant species, treatment duration, or UV dose), a pairwise Z‐test was applied. In cases of multiple subgroup comparisons, an appropriate Bonferroni correction was applied to account for the increased risk of false positives. All statistical analyses were performed using the Comprehensive Meta‐Analysis software (Biostat, Englewood, NJ, USA).

## 3 Results

### 3.1 Data Set Description

A systematic screening of the literature yielded 201 case studies. Each case study provided data on at least one proxy of water use efficiency (WUE). Specifically, the data set includes 127 case studies on stomata‐based WUE (sWUE), 137 on transpiration‐based WUE (eWUE), and 54 on isotope‐based WUE (isWUE). The sWUE and eWUE data were further categorized according to the light intensity conditions during gas‐exchange measurements, categorized as saturating (sat) and non‐saturating (non) light intensities. This categorization yielded the following subsets: sWUEnon – 50 studies, sWUEsat – 77 studies, eWUEnon – 56 studies, eWUEsat – 81 studies.

Most case studies (96) were conducted under field conditions, while 69 were performed in semi‐controlled greenhouse environments and 36 under controlled growth chamber conditions (Supporting Figure S3). Field studies were predominantly located in the subtropical zone of the Northern Hemisphere (approximately 20–35° latitude), whereas studies in the temperate and boreal zones (approximately 40–60° latitude) were substantially less well represented. The High Arctic, a region experiencing one of the most substantial relative increases in UV‐B radiation due to stratospheric ozone thinning, was represented by only two case studies on *Vaccinium uliginosum* (Boesgaard et al. 2012). No studies investigated the effects of UV on WUE in the tropical zone, which is characterized by the highest UV doses (Supporting Figure S4).

The data set comprises case studies on various plant growth forms, including herbs (78), grasses (35), shrubs (32), broadleaved trees (43), and coniferous trees (13). Of the case studies, 62 were categorized as long‐term (UV treatment > 90 days), 71 as short‐term (UV treatment ≤ 30 days), and 68 as midterm. The data set includes notable long‐term studies, such as a 5‐year UV‐B exclusion study on *Vaccinium uliginosum* (Boesgaard et al. 2012), a 5‐year UV‐B supplementation study on five broadleaved tree species (ash, birch, lime, oak, and sycamore) (Keiller and Holmes 2001), and a 3‐year UV‐B supplementation study on the coniferous species *Picea asperata* (Duan et al. 2011). In contrast, the shortest studies included were 2‐ and 4‐day chamber experiments on *Arabidopsis thaliana* (Ormrod et al. 1997). Further details of the compiled data set can be found in Supporting Table S1. In addition, Supporting Figure S4 illustrates the locations of field experiments included in the meta‐analysis. Among these, 26 case studies reported values of instantaneous gas‐exchange‐based sWUE and eWUE, four field studies provided data on all three WUE proxies (stomatal, transpirational, and isotopic), and only two field studies reported isotopic WUE.

### 3.2 Meta‐Analysis and Quantitative Assessment of UV Effects

**Figure 1:**
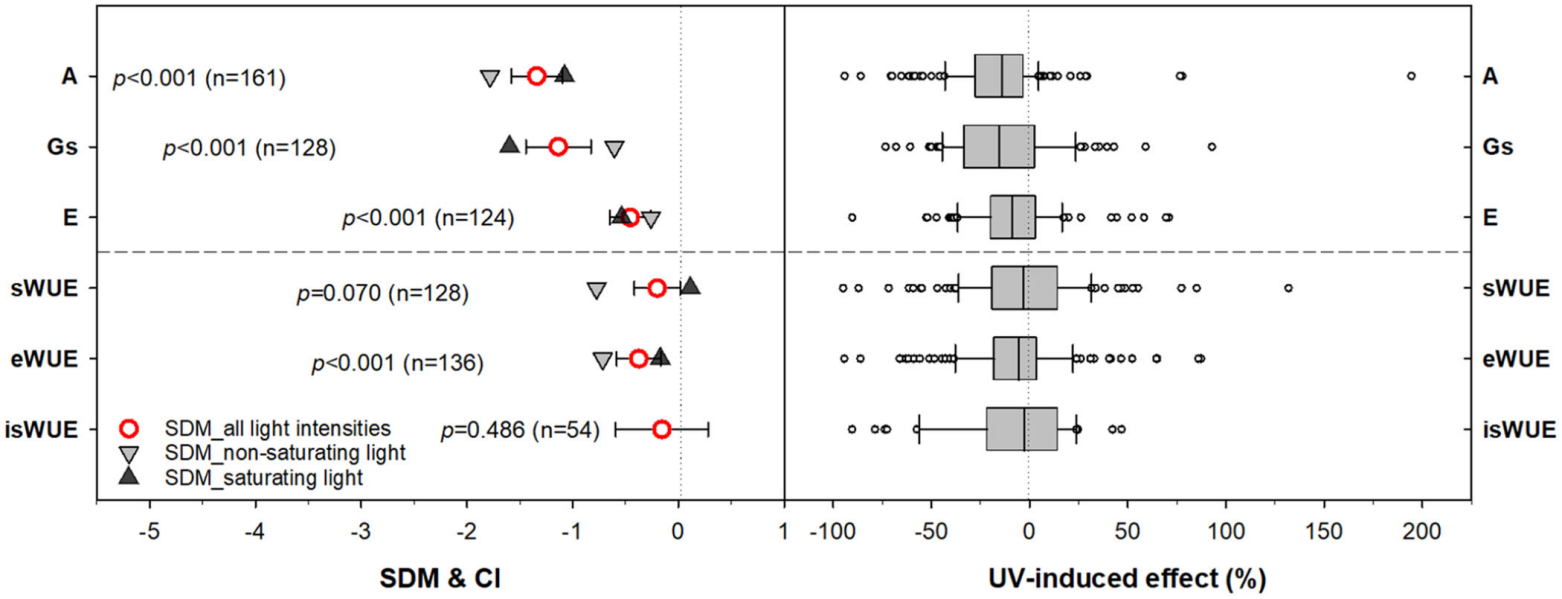
The overview of UV effects on CO_2_ assimilation rate (*A*), stomatal conductance (*G*s), transpiration rate (*E*), and water use efficiency calculated on the basis of *G*s (sWUE; *A*/*G*s), *E* (eWUE; *A*/*E*), and carbon stable isotope ratio (isWUE). Left panel: Meta‐analysis significance of UV‐induced effects: SDM, standard difference in means; 95% CI, 95% confidence interval. The numbers indicate the number of case studies included in the meta‐analysis. Right panel: Quantitative changes induced by UV radiation: vertical bars = medians, boxes = inter‐quartile range (IQR), whiskers = 5th and 95th percentiles, points = outliers identified.

A random‐effects model was applied to assess the variability in UV radiation effects across the included studies (Figure 1). This approach, in combination with the *p*‐value, enabled evaluation of the statistical significance of UV‐induced changes in the three categories of WUE, as well as the gas‐exchange parameters that determine these WUE values.

UV treatment resulted in significant (*p* < 0.001) negative effects on net photosynthesis (*A*), stomatal conductance (*G*s), and transpiration rate (*E*), with relative median declines of 14.0%, 15.4%, and 9.0%, respectively. The meta‐analysis also revealed a decline in all WUE proxies in UV‐treated plants, with median reductions ranging from 2.6% to 5.6%. However, the decline in isWUE, reflecting long‐term integrative acclimation, was only 2.6% and not statistically significant (*p* = 0.486). The decline in eWUE was significant (*p* < 0.001), while the decline in sWUE (*p* = 0.070) was not statistically significant but suggested a trend in instantaneous physiological changes. Field studies reporting multiple WUE proxies showed similar patterns. For example, UV‐B treatment induced declines in sWUE of 42.8% for *Arabidopsis thaliana* (Lake et al. 2009), 23.5% and 29.8% for *Morus alba* (Chen et al. 2016), and 35.0% for *Glycine max* (Chen et al. 2003). However, corresponding declines in isWUE were notably smaller, amounting to 26.2%, 12.6%, 4.4%, and 14.4%, respectively.

### 3.3 Variability in UV Effects

**Figure 2:**
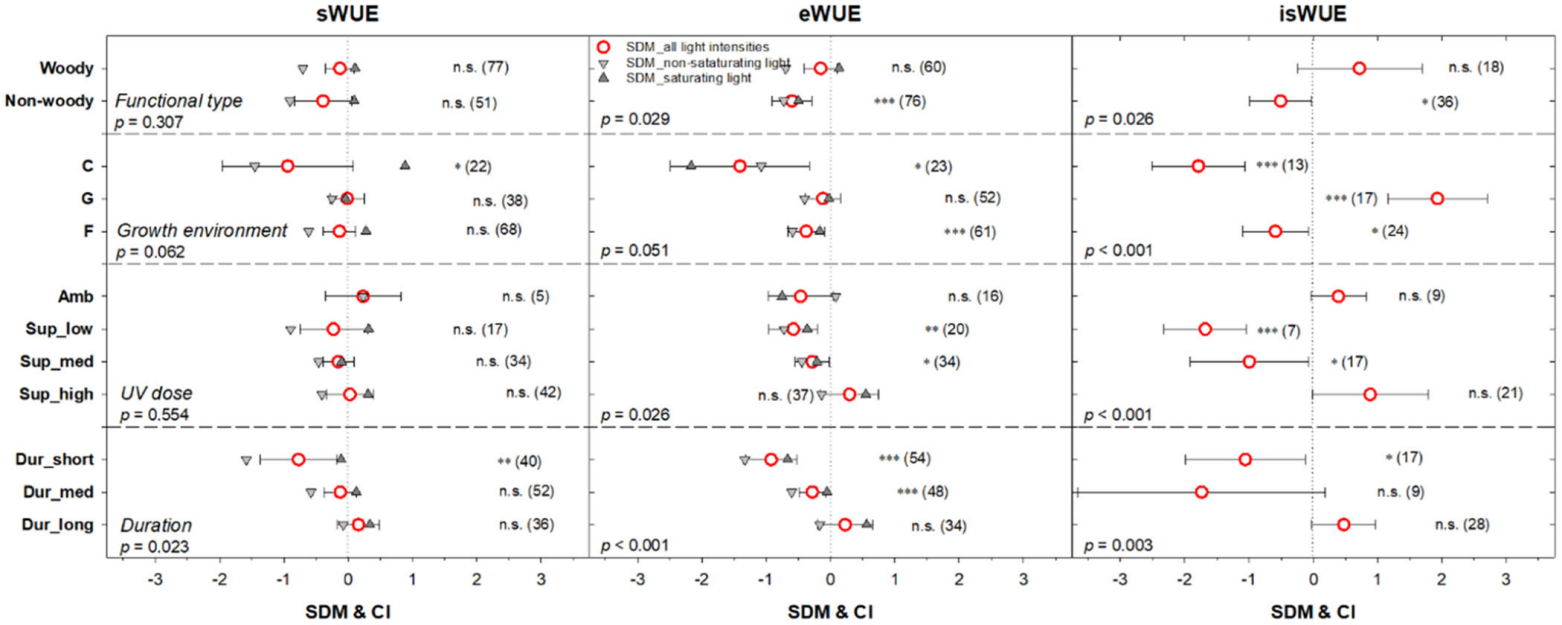
A detailed analysis of UV effects on water use efficiency based on stomatal conductance (sWUE), transpiration rate (eWUE), and abundance of stable carbon isotopes (isWUE). Symbols represent standard difference in means (SDMs), with error bars indicating 95% confidence intervals (CIs). Data are categorized by plant functional groups (Woody vs. Non‐woody), growth environment (C – growth chamber, G – greenhouse, F – field conditions), applied biologically effective UV dose in kJ m^–2^ day^–1^ (Amb – ambient UV intensity; supplementary doses: Sup_low – below 5 kJ m^–2^ day^–1^; Sup_med – 5–10 kJ m^–2^ day^–1^; Sup_high – above 10 kJ m^–2^ day^–1^, and UV treatment duration (Dur_short ≤ 30 days; Dur_med 30–90 days; Dur_long > 90 days). Asterisks denote statistical significance of UV effects within each group: n.s. – *p* > 0.05, * – 0.05 ≥ *p* > 0.01; ** – *p* ≤ 0.01; *** – *p* ≤ 0.001. Numbers in brackets indicate the count of case studies analysed per group. In addition, numerical *p*‐values indicate statistically significant differences within tested categories. For detailed analysis of UV effects on CO_2_ assimilation rate (*A*), stomatal conductance (*G*s), and transpiration rate (*E*), see Figure 4. [Color figure can be viewed at wileyonlinelibrary.com]

Responses of sWUE, eWUE, and isWUE were analysed based on plant functional type (woody vs. non‐woody plants), growth environment (chamber, greenhouse, or field), applied supplementary UV dose in kJ m^–2^ day^–1^ (Sup_low, Sup_med, or Sup_high), and duration of the UV treatment (Dur_short, Dur_med, or Dur_long) (Figure 2). Notably, all three WUE proxies exhibited consistent response patterns within these groups. For instance, non‐woody plants showed greater sensitivity to UV treatment across all WUE proxies compared to woody plants, although the difference was not statistically significant for sWUE (*p* = 0.307). Plants grown in growth chambers displayed substantially more negative UV effects on WUE than those grown under field conditions, which are characterized by exposure to the full sunlight spectrum. UV effects on WUE tended to diminish with increasing UV dose. This is particularly evident for isWUE (*p* < 0.001) and to a lesser extent for eWUE (*p* = 0.026), but was negligible for sWUE (*p* = 0.554). The duration of UV treatment also has a significant role in modulating effects on WUE. Short‐term treatments ( ≤ 30 days) induced significant negative impacts on all three WUE proxies, whereas these effects were negligible in long‐term studies ( > 90 days).

**Figure 3:**
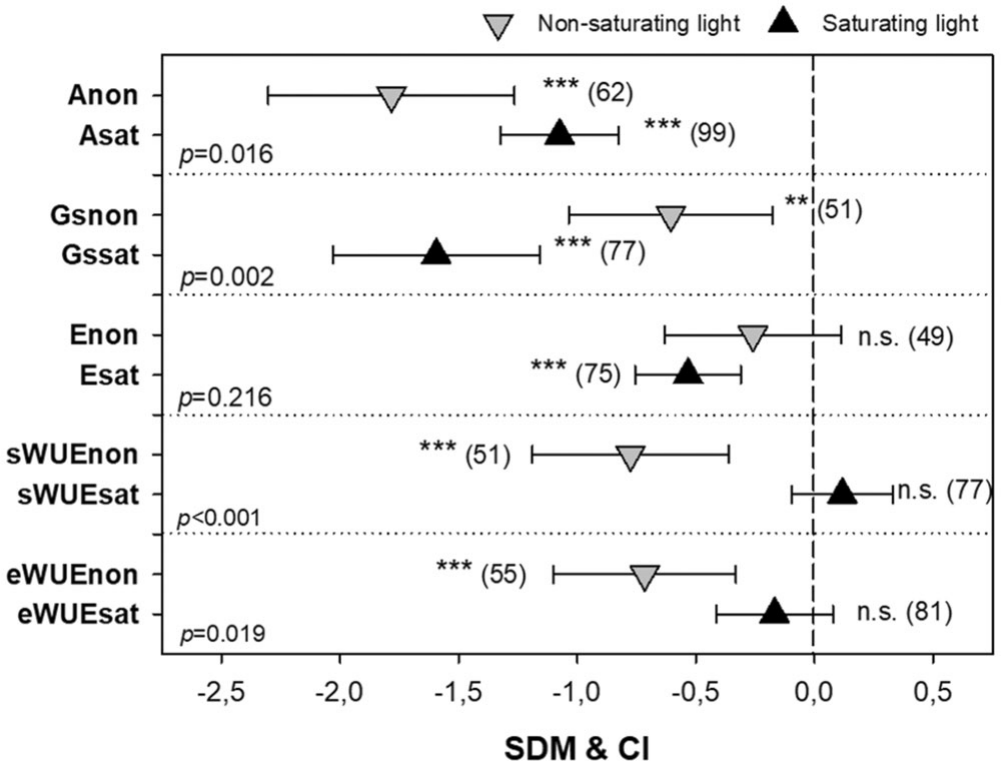
Meta‐analysis of UV‐induced effects on CO_2_ assimilation rate (*A*), stomatal conductance (*G*s), transpiration rate (*E*), stomata‐based water use efficiency (sWUE; *A*/*G*s), and transpiration‐based water use efficiency (eWUE; *A*/*E*). Symbols represent standard differences in means (SDMs) for non‐saturating light conditions (light grey triangles; non) and saturating light conditions (dark grey triangles; sat), with error bars showing 95% confidence intervals (CIs). Asterisks indicate the statistical significance of UV effects within each group: n.s. – *p* > 0.05, * – 0.05 ≥ *p* > 0.01; ** – *p* ≤ 0.01; *** – *p* ≤ 0.001. Numbers in brackets show the number of case studies analysed per group. *p*‐values on the left indicate statistically significant differences between SDM values under saturating ( > 1000 µmol m^–2^ s^–1^) and non‐saturating intensities of photosynthetically active radiation (PAR).

Substantial differences in SDM values for all gas‐exchange parameters were observed when these were measured under either saturating or non‐saturating light intensities (light *vs.* dark grey triangles in Figure 1). Detailed analysis (Figure 3) showed that the UV effect on both sWUE and eWUE was nonsignificant (*p* > 0.05) under saturating ( > 1000 µmol m^–2^ s^–1^) intensities of photosynthetically active radiation (PAR), but highly significant (*p* < 0.001) under non‐saturating PAR intensities. This discrepancy arises from significantly stronger UV effects on photosynthetic CO_2_ uptake under non‐saturating light, whereas the impact of light conditions on stomatal conductance was smallest under these light conditions, whilst the effect on transpiration was negligible.

**Figure 4:**
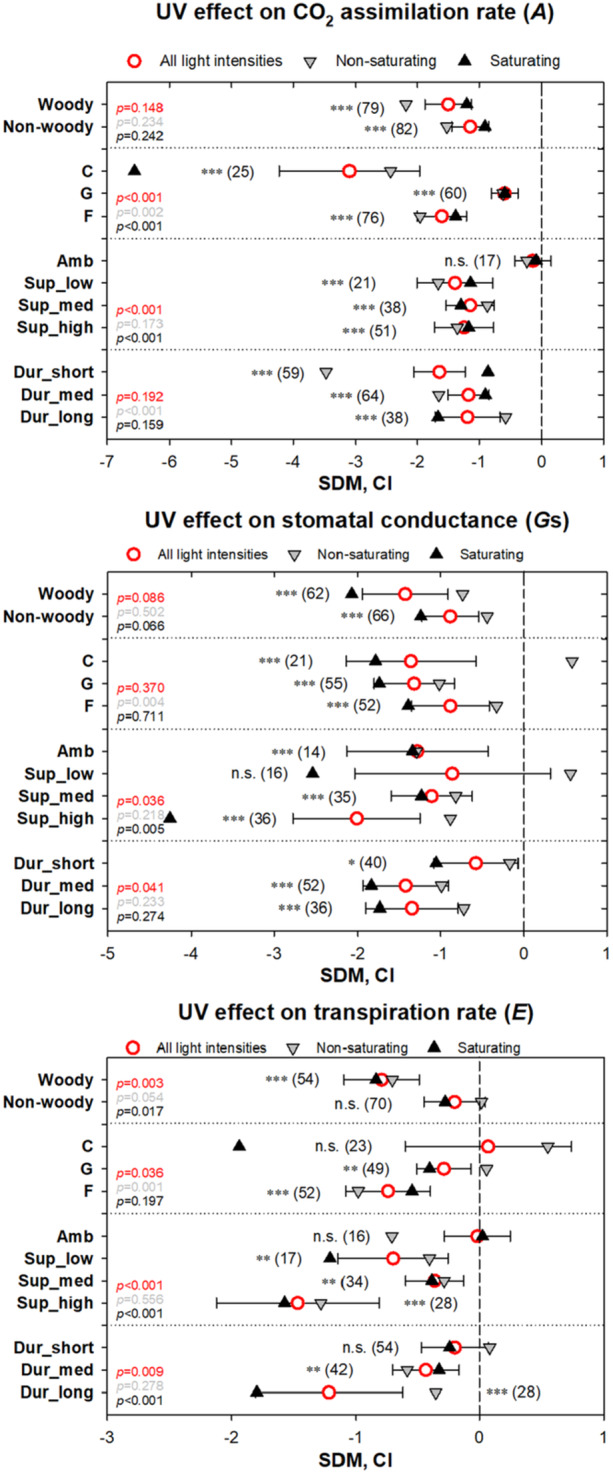
Analysis of instantaneous UV effects on CO_2_ assimilation rate (*A*; upper panel), stomatal conductance (*G*s; middle panel), and transpiration rate (*E*; bottom panel) determined via gas‐exchange techniques. Symbols represent the standard difference in means (SDMs), with error bars showing 95% confidence intervals (CIs). Red circles indicate SDM values for all studies regardless of light intensity, while light grey and dark grey triangles represent SDM values for studies conducted under non‐saturating and saturating light intensities, respectively. All parameters are further categorized by plant functional groups (Woody vs. Non‐woody), growth environment (C – growth chamber, G – greenhouse, F – field conditions), applied biologically effective UV dose in kJ m^–2^ day^–1^ (Amb – ambient UV intensity (exclusion type of UV experiments), supplementary doses (Sup_low – below 5 kJ m^–2^ day^–1^; Sup_med – 5–10 kJ m^–2^ day^–1^; Sup_high – above 10 kJ m^–2^ day^–1^), and UV treatment duration (Dur_short ≤ 30 days, Dur_med 30–90 days, Dur_long > 90 days). Asterisks denote the statistical significance of UV effects within each group: n.s. – *p* > 0.05, * – 0.05 ≥ *p* > 0.01; ** – *p* ≤ 0.01; *** – *p* ≤ 0.001. Numbers in brackets indicate the number of case studies analysed per group. Additionally, *p*‐values highlight statistically significant UV effects within tested categories for all light conditions (red), non‐saturating light conditions (light grey), and saturating light intensities (dark grey). [Color figure can be viewed at wileyonlinelibrary.com]

A detailed breakdown of UV‐B‐induced changes in the predictors of sWUE and eWUE—*A*, *G*s, and *E*—reveals distinct response patterns across different conditions (Figure 4). The impact of UV‐B radiation on *A* was similar for woody and non‐woody plant species, with a stronger effect under non‐saturating compared to saturating light intensities. The pronounced effects of UV‐B radiation on primary photochemical reactions (i.e. under non‐saturating light) were particularly evident in short‐term treatments, while the effects weakened over longer‐term treatments, indicating the photosynthetic machinery's capacity to acclimate to enhanced UV‐B doses. Notably, exclusion UV‐B studies did not reveal a significant effect on *A*, while supplemental UV‐B studies demonstrated a significant negative impact, irrespective of the UV‐B dose applied. In contrast, the negative effects of UV‐B radiation on *G*s significantly increased with increasing supplemental UV‐B doses. Moreover, *G*s was less affected by short‐term treatments compared to medium‐ and long‐term treatments. This long‐term acclimation trend in *G*s was even more pronounced for *E*. UV‐B radiation induced significantly (*p* = 0.003) stronger reduction in *E* in woody plants than in non‐woody plants. This trend was also observed for *G*s but was not statistically significant (*p* = 0.086). In contrast to *G*s, the effect of UV‐B radiation on *E* was substantially influenced by the growth environment, showing a strong decrease under field conditions, a less pronounced effect under greenhouse conditions, and a negligible effect under growth chamber environments. These findings suggest a possible interaction between UV‐B radiation and other environmental drivers, influencing the total amount of water transpired through plant leaves.

### 3.4 Mechanisms of UV‐Induced Changes in sWUE and eWUE

**Figure 5:**
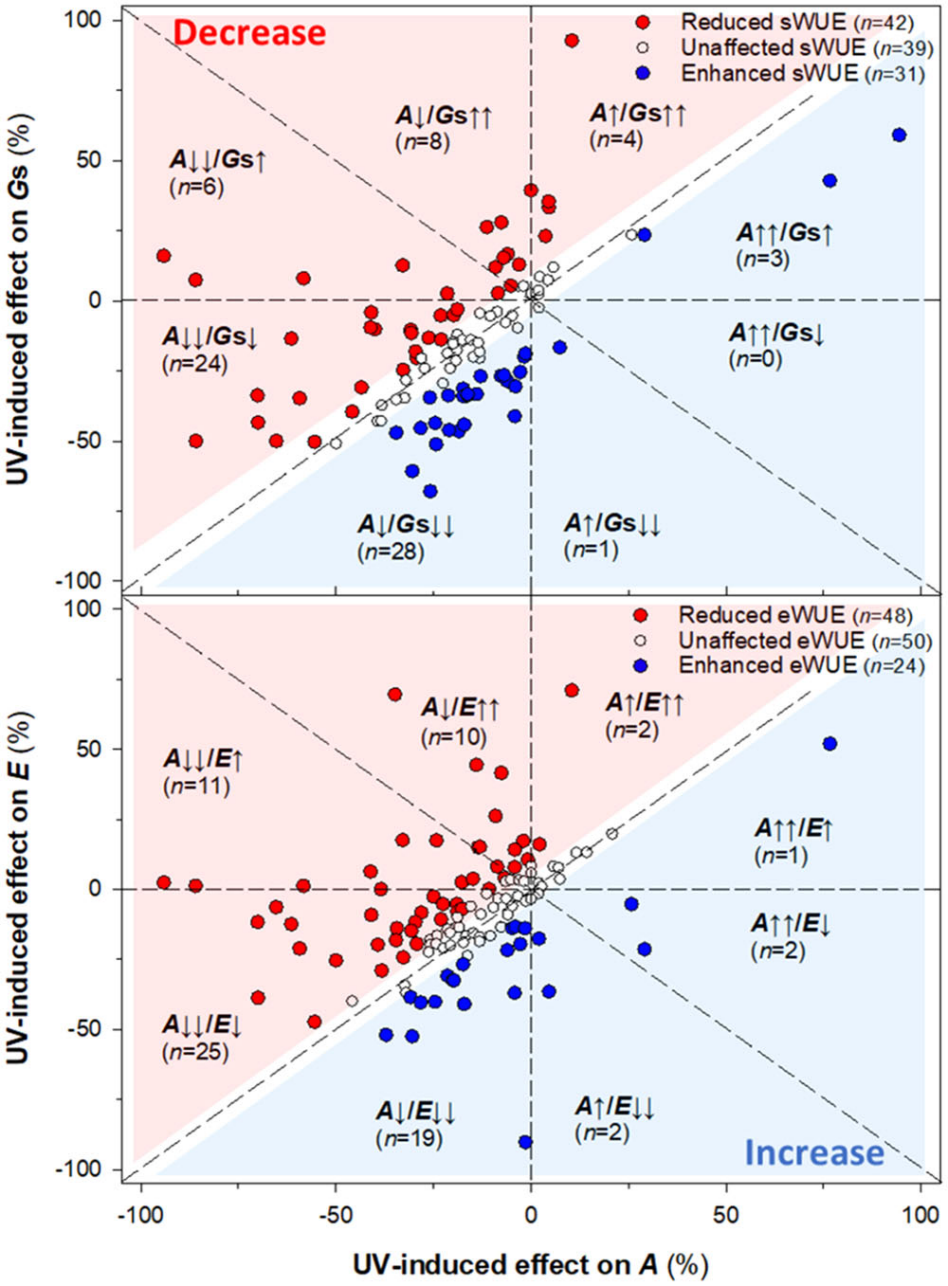
Relationship between UV‐induced effects on CO_2_ assimilation rate (*A*) and stomatal conductance (*G*s; upper panel) or transpiration rate (*E*; lower panel). White circles indicate case studies where UV effects on water use efficiency (WUE) ranged between −10% (decrease) and +10% (increase), reflecting proportional changes in *A* and *G*s (sWUE) or *A* and *E* (eWUE). Red (blue) circles represent case studies showing a UV‐induced decrease (increase) in WUE due to disproportional changes in *A*/*G*s or *A*/*E*. Arrows depict increase (↑) or decrease (↓) in each parameter within all segments of the graph, with double arrows indicating stronger UV effects. The total number of case studies per category is indicated by *n*‐values. [Color figure can be viewed at wileyonlinelibrary.com]

Theoretically, WUE remains unchanged if UV‐induced changes in *A* and *G*s (or *E*) are proportional. Using a threshold of ±10% as the criterion for a substantial UV effect on WUE, we found that 35% of case studies reported negligible effects on sWUE. Similarly, 41% of case studies reported negligible effects on eWUE. When changes in *A* and *G*s (or *E*) are not proportional, WUE either increases or decreases, depending on the specific combination of their changes. As shown in Figure 5, case studies reporting declines in sWUE (38%) and eWUE (39%) were more frequent than case studies reporting enhancements (28% for sWUE and 20% for eWUE). Figure 5 also illustrates the two primary mechanisms driving the negative UV effects on sWUE and eWUE. The dominant mechanism involves a greater reduction in photosynthesis (*A*) compared to stomatal conductance (*G*s) or transpiration rate (*E*). Additionally, a substantial proportion of case studies reported UV‐stimulated increases in *G*s and *E* without a corresponding increase in *A*. These findings indicate that UV radiation often impairs the CO_2_ fixation process more than it disrupts water regulation, leading to reduced sWUE. Conversely, increases in sWUE under UV radiation typically result from more efficient stomatal closure relative to the reduction in photosynthesis. Studies reporting substantial UV‐induced increases in *A* were rare. Similar trends were observed for eWUE. However, unlike sWUE, we identified four case studies where eWUE substantially increased due to an upward trend in *A* combined with a downward trend in *E*. Notably, these studies on *Acorus calamus* (Kumari et al. 2009) and *Vaccinium corymbosum* cv. Legacy (González‐Villagra et al. 2020) reported contrasting UV‐B‐induced effects on sWUE and eWUE. While sWUE consistently decreased in these cases, eWUE increased. Such contrasting findings may indicate incorrect determination of one of the gas‐exchange parameters or suggest a strong interactive effect of environmental drivers on transpiration rate. For instance, relatively high stomatal conductance may not always result in a proportional increase in transpiration.

## 4 Discussion

A timely question in the context of climate change adaptation is how WUE will be affected by changes in various environmental variables, including UV‐exposure (Hatfield and Dold 2019). Previous studies have highlighted (Jansen et al. 2022) the commonalities between plant responses to drought and UV‐B, whilst a recent meta‐analysis highlighted negative effects of UV‐B radiation on stomatal aperture and size (Ač et al. 2024). An unresolved, but critical, question is whether the UV‐induced closure of stomata will also result in an improved WUE of plants, and even increased drought resilience. Thus far, individual studies of WUE in UV‐B exposed crops have yielded widely diverging results (e.g. He et al. 2014 vs Wang et al. 2024). Indeed, the interpretation of the results of individual UV‐B manipulation experiments is beset with methodological difficulties relating, amongst others, to the choice of lamps, exposure chambers, optical filters and timing of exposure (Aphalo et al. 2012). In the present analysis of the literature, those studies where such matters were insufficiently described were filtered out. Most of the retained papers are from the ‘modern’ era, i.e. published since 2000 (Supporting Figure S2), an era of increased awareness of methodological issues (Aphalo et al. 2012). The result is a robust data set suitable for detailed meta‐analysis. The data set reflects the diversity of responses that plant species and functional types show to UV‐B exposure. It might be anticipated that differences in plant UV‐responses would occur between taxonomic or functional plant groups, associated with differences in UV‐resistance. However, no significant differences in UV‐response were noted across the functional groups included in the database.

### 4.1 UV‐B Usually Reduces Photosynthesis

In the present analysis, an overwhelming (81%) proportion of the studies shows that UV‐B enriched light decreases the rate of photosynthesis, especially when measured under non‐saturating irradiance. *A*sat primarily reflects the activity and abundance of the Rubisco enzyme controlling CO_2_ assimilation during Calvin cycle (von Caemmerer and Quick 2000), whereas *A*non is mainly limited by the rate of linear electron transport on the thylakoid membrane. Thus, the large reduction of photosynthesis under non‐saturating irradiance is consistent with a UV impact on the primary photochemical reactions of photosynthesis, including light energy absorption and electron transport leading to ATP and NADPH production, as others have found (see earlier reviews by Teramura and Sullivan 1994; Kataria et al. 2014). The relatively modest UV effect under saturating light conditions further strengthens the overall conclusion that PSII is a primary target for UV‐B radiation.

The observed UV‐B effects on photosynthesis seemingly contradict the broadly accepted conclusion that UV‐B is predominantly a regulator of plant physiology, rather than a stressor (Fiscus and Booker 1995; Allen et al. 1998; Jansen and Bornman 2012). However, more detailed analysis reveals that when the data are grouped according to their respective exposure methodologies, the strong UV‐B effect on photosynthesis is seen only in those experiments where UV is applied as supplemental radiation. Conversely, where the experimental protocol was to exclude ambient UV irradiance under field conditions, the treatment effect on photosynthesis was not statistically significant (Supporting Figure S5). Thus, UV effects are associated with above‐ambient and/or artificial UV‐B exposure levels. Negative effects of UV on photosynthesis are particularly associated with chamber experiments, and inhibitory UV effects can most likely be explained by an artificially high UV to PAR ratio (Deckmyn and Impens 1997). Furthermore, under indoor conditions, inhibition of photosynthesis may also be aggravated by the absence of longer wavebands of UV (UV‐A, 315–350 nm). UV‐A is intense in sunlight but usually lacking or low in growth chambers and greenhouses. Apart from the role of UV‐A and blue light in photorepair (Banaś et al. 2020), there may also be ‘cross‐talk’ between UV‐A and UV‐B photoreceptors (as suggested by Rai et al. 2020, 2021), driving further repair and protection responses. Understanding this balance between damage, repair and acclimation responses is one of the continuing challenges for UV research. Surprisingly, the present study found no evidence that UV‐induced acclimation responses protect photosynthesis. In fact, the extent of the damage caused by supplemental UV‐B was similar irrespective of whether the UV treatment was of short, medium or prolonged duration (Figure 4 and Supporting Figure S5). These data indicate the importance of constitutive UV protection. All in all, the meta‐analysis emphasises the importance of environmentally realistic experimentation, and this is particularly important where data are used to assess environmental responses and/or to model global carbon fluxes.

### 4.2 UV‐B Usually Reduces Leaf Stomatal Conductance (*G*s) and Transpiration (*E*)

Overall, the effects of UV‐B enriched light on leaf stomatal conductance and transpiration are significantly negative (Figure 1), irrespective of the duration and exposure protocol of the experiments (Figure 4). It is likely that these negative effects on stomatal conductance and transpiration comprise a slight underestimate as measurements were predominantly performed in cuvettes, in the absence of UV radiation, so it can be speculated that some reopening of stomata may have occurred following cessation of UV‐treatment, and handling of leaves (Lawson and Vialet‐Chabrand 2019). However, at present there are no experimental data on the dynamics of stomatal opening and closure after UV treatment that can help quantify this underestimate. The overall negative UV effect on leaf stomatal conductance and transpiration is consistent with previous analyses showing a positive association between UV and drought responses (Jansen et al. 2022) and a UV‐mediated decrease of stomatal conductance, stomatal aperture and stomatal size (but not stomatal density, which increased in UV‐exposed plants) (Ač et al. 2024). UV‐B‐mediated stomatal closure has been identified as UVR8‐mediated, involving ethylene synthesis and signalling (Ge et al. 2020). Similarly, the UV‐B‐mediated increase in stomatal density is UVR8‐controlled, with a UVR8‐deficient mutant revealing a decrease in stomatal density following UV‐B exposure (Wargent et al. 2009). UVR8‐mediated increases in stomatal density are paralleled by decreases in aperture (Ač et al. 2024), triggering questions concerning the functional role of these seemingly contradictory changes. The current results suggest that, on balance, decreases in stomatal conductance, aperture, and size negate the effect of increased stomatal density with overall clear decreases in leaf transpiration and conductance. This finding is consistent with earlier studies showing that stomatal conductance, rather than stomatal density, is the main determinant of leaf transpiration (Tricker et al. 2005).

There are minor differences in the effects of UV radiation on conductance versus transpiration. In this study, transpiration (*E*) was found to be generally reduced, except in chamber or short‐term experiments and in ambient UV exposure experiments. This variable response can be speculated to be due to diverse, and sometimes unreliable methods of measuring transpiration, in combination with variability in vapour pressure deficit and other environmental parameters that will affect transpiration rates (e.g. wind). The strong dependence of transpiration on external environmental conditions must be kept in mind when investigating the UV effect on water use efficiency in the future. In contrast, stomatal conductance is a widely accepted measure of the physiological status of the stomata (Cowan 1978). However, measurements of stomatal conductance fail to convey potential effects on water loss. Here, the effects of UV‐B on leaf stomatal conductance are negative, presumably due to UVR8‐dependent stomatal closure (Ge et al. 2020). A decline in stomatal conductance, while potentially conserving water, will inevitably cause an increase in leaf temperature with an associated increase in the leaf‐to‐air vapour pressure difference (VPD). In turn, this increase in vapour pressure deficit can increase transpiration (or at least moderate the UV‐induced decrease in transpiration) (Marchin et al. 2016; Martínez‐Lüscher et al. 2013). The increase in leaf temperature may also directly affect stomata by increasing stomatal conductance, and this would result in an increase in transpiration (Urban et al. 2017). Notwithstanding these interactive, and even opposing, effects, the current data set overwhelmingly shows that UV‐B has a negative effect on both conductance and transpiration.

In the present analysis, the smallest UV effects on leaf stomatal conductance are found in short‐term studies. As a short‐term study would preclude UV‐induced acclimatory changes in leaf morphology, including stomatal size and density, it is likely that the short‐term effect is dominated by rapid changes in stomatal aperture. The somewhat larger UV effects on stomatal conductance in medium and long‐term studies may imply a role for additional, slower morphological adjustments, such as a decrease in stomatal size (Ač et al. 2024).

### 4.3 UV‐B Rarely Improves Water use Efficiency

It has been proposed that UV‐B radiation may improve WUE by its influence on stomatal aperture and leaf gas exchange (Gitz III et al. 2005; Ač et al. 2024). Yet, the current study shows that a UV‐B‐induced increase in WUE is sometimes observed, but in most cases, it is not the case. In both chamber and short‐term experiments, UV‐B treatment reduces the WUE substantially. Conversely, in long‐term studies, there is no effect of UV‐B treatment— neither negative nor positive. As WUE represents the ratio of carbon uptake gain to water loss, changes in WUE can be caused by UV effects on either photosynthesis or stomatal conductance and transpiration. Thus, a lack of UV effect on WUE can be due to a lack of effect on either parameter, or alternatively, similar effects on these physiological parameters. To explore this, data were presented as positive or negative relative (%) effects on either parameter, in a scatterplot (Figure 5). The majority (27 of 46) of case studies showed a proportionate reduction in both photosynthesis and conductance so that WUE was essentially unaffected by UV‐B (open points in Figure 5). In 31 of 116 cases, WUE decreased moderately (−30% to −10%), while in 16 of 116 cases, it declined strongly (< −30%), and this was overwhelmingly attributed to a larger decrease in photosynthesis compared to conductance and/or transpiration. A minority of studies reported a strong ( > 30%) increase in WUE caused by a strong decline in stomatal conductance or transpiration without a proportionate change in photosynthesis.

Negative effects of UV‐treatment on WUE in growth chamber and short‐term experiments are most substantial, and this can be attributed to relatively large effects on photosynthesis under these experimental conditions. Thus, UV radiation impairs the CO_2_ fixation process more than it impacts water loss. However, as argued before, the decrease in photosynthesis is largely associated with above ambient UV‐B doses and/or artificial growth conditions (i.e. chambers). Consistently, field studies (and the partially overlapping category of long‐term studies) show no, or very small, effects on WUE. In fact, substantial numbers of field studies report positive effects of UV‐B exposure on WUE. For example, both Sullivan et al. (2003) and Lou et al. (2016) report a positive effect of UV‐B treatment on WUE under field conditions. Yet, also some growth chamber studies report positive UV‐B effects on WUE (Poulson et al. 2006; Gupta and Prasad 2021). At present, it is not clear whether these positive outcomes of UV treatment on WUE are associated with particular experimental details, such as the length of the UV treatment, the UV dose, or the choice of plant species. Nevertheless, this is an important question as the identification of parameters associated with an increase in WUE can assist in adaptation of crops to drier weather conditions.

### 4.4 A Comparison of Methods Used to Assess WUE

In the current analysis, WUE data were grouped according to three distinct methods of calculation (Figures 1, 2). The calculation of eWUE and sWUE follows widely used methods, based on the ratio of net photosynthesis and transpiration or stomatal conductance, respectively. Gas exchange methods provide instantaneous values of WUE (eWUE and sWUE) at specific moments, whether measured in the field or in a growth chamber. In contrast, the stable isotope method (isWUE) integrates plant responses over longer time scales (Farquhar et al. 1989; Seibt et al. 2008), reflecting cumulative physiological adjustments to UV‐B radiation. The instantaneous WUE (eWUE and sWUE) is highly sensitive to environmental conditions (weather, time of day and so on) during the actual measurement, which may increase uncertainty in interpreting UV‐induced effects and hamper the comparison of various species or case studies, especially when variables influencing stomatal conductance, such as VPD, light intensity, or temperature, are not reported. From this perspective, a greater emphasis should be placed on the isotopic method as this integrates WUE over a substantial period of leaf development and should therefore yield more robust results enabling comparisons across a wide spectrum of case studies, plant species and biomes. In this study, it was found that there is a broad, overall similarity in the patterns of response across the three WUE calculation methods, including, for example, negative effects on WUE under growth chamber conditions and in short‐term studies, and a lack of UV effect in long‐term studies (Figure 2). Interestingly, the isWUE proxy reveals a positive UV effect on WUE under greenhouse conditions, an effect not detected using the instantaneous eWUE or sWUE proxies.

The origin of the slightly different results for sWUE, eWUE and isWUE is likely related to the instantaneous nature of sWUE, eWUE, compared to time‐integrated assessment of isWUE. Additionally, it should be noted that the functional basis of the three WUE proxies differs. While eWUE reflects carbon assimilation relative to transpired water, sWUE and isWUE relate carbon gain to stomatal conductance without directly quantifying water loss. Transpiration rates tend to increase in dry atmospheres with high VPD and decrease in wet atmospheres with low VPD. However, sWUE and isWUE do not necessarily reflect this VPD‐driven phenomenon (Ehleringer et al. 1993; Treydte et al. 2024). Several studies have shown that high VPD can increase transpiration rates even when stomata are partially or fully closed, leading to significant water loss (Marchin et al. 2016; Leonardi et al. 2000; Strange et al. 2023). Therefore, it could be argued that sWUE and isWUE may not fully capture plant water balance, which is more directly determined by total transpired water rather than stomatal conductance alone (Guerrieri et al. 2019). Accordingly, recent studies suggest further refinement of isWUE calculations to account for atmospheric dryness (Strange et al. 2023; Pernicová et al. 2024; Treydte et al. 2024). Overall, the data in this study show that different WUE proxies can yield slightly different results. This emphasises that to obtain a comprehensive assessment of WUE responses to UV‐B radiation, the use of multiple WUE proxies is recommended.

### 4.5 Relevance to Field Conditions

UV‐B radiation and drought induce similar, partially overlapping responses in plants, including decreases in stomatal conductance and transpiration (Jansen et al. 2022). Therefore, it can be hypothesised that UV‐B can protect plants against drought. Consistently, it has been shown that exposure of greenhouse‐grown lettuce (*Lactuca sativa*) to solar UV‐B radiation conferred protection against transplantation stress (Wargent et al. 2011). The underlying mechanism is likely multifaceted but given that transplanting stress typically involves a temporary imbalance between water uptake and loss, a role for decreased water loss might be hypothesised. Similarly, Crestani et al. (2023) showed that ‘priming’ of plantlets with a low dose of UV‐B helps to protect the plantlets from drought when they are transplanted from closed tissue‐culture conditions to soil. The current study shows that reductions in transpiration and stomatal conductance are closely associated with a yield penalty, i.e. decreases in photosynthesis, and an overall decrease in WUE. Nevertheless, UV‐B induced decreases of water‐loss are likely to be of interest to the horticultural industry, especially to prevent short‐term drought stress (e.g. transplanting stress and other stresses encountered during long‐distance transport) when the yield penalty will be correspondingly minor. The findings of this paper will also be of interest in the context of climate change adaptation, and especially crop acclimation to short episodes of drought, accompanied by high UV radiation. Under these conditions, UV‐exposure will decrease water loss and facilitate plant survival, while effects on long‐term productivity are likely to be modest.

Consequently, including UV‐B exposure data in climate change modelling studies is likely to improve the accuracy of climate‐vegetation feedback.

## 5 Conclusions

This meta‐analysis explores whether reported decreases in stomatal aperture and transpiration in UV‐exposed plants enhance WUE, a timely question in the context of climate change. Our findings confirm that UV‐B exposure decreases stomatal conductance and transpiration; however, this effect is counterbalanced by an equally strong or even stronger decline in photosynthetic rate, especially under non‐saturating light conditions. As a result, WUE is either unchanged or decreased, depending on the methodology used for the WUE assessment. Notably, isotope‐based WUE proxies, which evaluate carbon and water fluxes over longer time periods, indicate no significant UV‐B effect on WUE, whereas gas exchange‐based proxies, which capture instantaneous physiological responses, often show a decline. This discrepancy highlights the importance of appropriate methodological approaches for assessing plant water use efficiency. Furthermore, decreases in photosynthesis are only significant in studies where UV‐B is applied as supplemental radiation, whereas exclusion of ambient UV‐B under field conditions does not significantly affect photosynthesis and has neutral or even positive effects on WUE. These differences show that the impacts of UV‐B on WUE depend strongly on exposure conditions, duration, and plant acclimation potential.

These data also underscore the importance of long‐term field studies that integrate multiple environmental drivers to more accurately assess adjustments in plant water use efficiency in a changing world. Overall, this study challenges the assumption that UV‐B‐induced stomatal closure improves WUE by reducing water loss. Instead, the results indicate a complex interplay between UV‐B exposure, photosynthesis, and water‐use regulation, which may be further modulated by interactive effects with environmental stressors such as drought and elevated temperatures.

## Conflicts of Interest

The authors declare no conflicts of interest.

## Supporting information

References SupplementaryTableS1.

SupplementaryTable S1 alphabetical.

SuppMat.

## Data Availability

The data that support the findings of this study are openly available in ASEP at https://doi.org/10.57680/asep.0619774 https://asep-portal.lib.cas.cz/.
