# Supplementary material for: A Global Meta‐Analysis of Water Use Efficiency Proxies Reveals That UV Radiation Decreases Transpiration Without Improving WUE
Source: Plant Cell Environ. 2025 May 28;48(9):6734–47. doi: 10.1111/pce.15643 (PMC12319297; doi:10.1111/pce.15643)
Supplement: Supplementary file 1 — References SupplementaryTableS1. [file PCE-48-6734-s003.docx]

**Reference list to Supplementary Table S1**

Basahi JM, Ismail IM, Hassan IA (2014) Effects of enhanced UV-B radiation and drought stress on photosynthetic performance of lettuce (*Lactuca sativa* L. Romaine) plants. *Annual Research & Review in Biology*, **4**, 1739–1756.

Bassman JH, Robberecht R, Edwards GE (2001) Effects of enhanced UV-B radiation on growth and gas exchange in *Populus deltoides* Bartr. ex Marsh. *International Journal of Plant Sciences*, **162**, 103–110.

Berli FJ, Alonso R, Bressan‐Smith R, Bottini R (2013) UV‐B impairs growth and gas exchange in grapevines grown in high altitude. *Physiologia Plantarum*, **149**, 127–140.

Boesgaard KS, Albert KR, Ro‐Poulsen H, Michelsen A, Mikkelsen TN, Schmidt NM (2012) Long‐term structural canopy changes sustain net photosynthesis per ground area in high arctic *Vaccinium uliginosum* exposed to changes in near‐ambient UV‐B levels. *Physiologia Plantarum*, 145, 540–550.

Chen M, Huang Y, Liu G, Qin F, Yang S, Xu X (2016) Effects of enhanced UV-B radiation on morphology, physiology, biomass, leaf anatomy and ultrastructure in male and female mulberry (*Morus alba*) saplings. *Environmental and Experimental Botany*, **129**, 85–93.

Chen T, Qiang W, An L, Wang X (2003) Effects of cadmium combined with ultraviolet-B radiation on growth, gas exchange, and stable carbon isotope value (δ^13^C) in soybean (*Glycine max* L.). In: *SPEI Proceedings* - Ultraviolet Ground-and Space-based Measurements, Models, and Effects II, Vol. 4896, pp. 210–218.

Chu R, Zhang QH, Wei YZ (2022) Effect of enhanced UV-B radiation on growth and photosynthetic physiology of *Iris tectorum* maxim. *Photosynthesis Research*, **153**, 177–189.

Correia CM, Pereira JMM, Coutinho JF, Björn LO, Torres-Pereira JM (2005) Ultraviolet-B radiation and nitrogen affect the photosynthesis of maize: a Mediterranean field study. *European Journal of Agronomy*, **22**, 337–347.

Correia CM, Torres-Pereira MS, Torres-Pereira JMG (1999) Growth, photosynthesis and UV-B absorbing compounds of Portuguese Barbela wheat exposed to ultraviolet-B radiation. *Environmental Pollution*, **104**, 383–388.

Del-Castillo-Alonso MÁ, Diago MP, Tomás-Las-Heras R, Monforte L, Soriano G, Martínez-Abaigar J, Núñez-Olivera E (2016) Effects of ambient solar UV radiation on grapevine leaf physiology and berry phenolic composition along one entire season under Mediterranean field conditions. *Plant Physiology and Biochemistry*, **109**, 374–386.

Dias MC, Pinto DC, Correia C, Moutinho-Pereira J, Oliveira H, Freitas H, Silva AMS, Santos C (2018) UV-B radiation modulates physiology and lipophilic metabolite profile in *Olea europaea*. *Journal of Plant Physiology*, **222**, 39–50.

Doupis G, Chartzoulakis K, Patakas A (2012) Differences in antioxidant mechanisms in grapevines subjected to drought and enhanced UV-B radiation. *Emirates Journal of Food & Agriculture* (EJFA), **24**, 607–613.

Duan B, Ran F, Zhang X, Zhang Y, Korpelainen H, Li C (2011) Long-term acclimation of mesophyll conductance, carbon isotope discrimination and growth in two contrasting *Picea asperata* populations exposed to drought and enhanced UV-B radiation for three years. *Agricultural and Forest Meteorology*, **151**, 116–126.

Duan B, Xuan Z, Zhang X, Korpelainen H, Li C (2008) Interactions between drought, ABA application and supplemental UV‐B in *Populus yunnanensis*. *Physiologia Plantarum*, **134**, 257–269.

Feng H-Y, Chen T, Xu S-J, An L-Z, Qiang W-Y, Zhang M-X, Wang X-L (2001) Effect of enhanced UV-B radiation on growth, yield and stable carbon isotope composition in *Glycine max* cultivars. *Acta Botanica Sinica*, **43**, 709–713.

Feng H, An L, Chen T, Qiang W, Xu S, Zhang M, ... Cheng G (2003) The effect of enhanced ultraviolet-B radiation on growth, photosynthesis and stable carbon isotope composition (δ^13^C) of two soybean cultivars (*Glycine max*) under field conditions. *Environmental and Experimental Botany*, **49**, 1-8.

Gaberščik A, Vončina M, Trošt T, Germ M, Björn LO (2002) Growth and production of buckwheat (*Fagopyrum esculentum*) treated with reduced, ambient, and enhanced UV-B radiation. *Journal of Photochemistry and Photobiology B: Biology*, **66**, 30–36.

Gitz III DC, Britz SJ, Sullivan JH (2013) Effect of ambient UV-B on stomatal density, conductance and isotope discrimination in four field grown soybean [*Glycine max* (L.) Merr.] isolines. *American Journal of Plant Sciences*, **4**, 100–108.

Gitz III DC, Liu-Gitz L, Britz SJ, Sullivan JH (2005) Ultraviolet-B effects on stomatal density, water-use efficiency, and stable carbon isotope discrimination in four glasshouse-grown soybean (*Glyicine max*) cultivars. *Environmental and Experimental Botany*, **53**, 343–355.

González-Villagra J, Marjorie RD, Alberdi M, Acevedo P, Loyola R, Tighe-Neira R, Arce-Johnson P, Inostroza-Blancheteau C (2020) Solar UV irradiation effects on photosynthetic performance, biochemical markers, and gene expression in highbush blueberry (*Vaccinium corymbosum* L.) cultivars. *Scientia Horticulturae*, **259**, 108816.

Gupta D, Prasad SM (2021) Priming with 5-aminolevulinic acid (ALA) attenuates UV-B induced damaging effects in two varieties of *Cajanus cajan* L. seedlings by regulating photosynthetic and antioxidant systems. *South African Journal of Botany*, **138**, 129–140.

Habibi G, Hajiboland R, Dehghan G (2010) Contrastive response of *Phlomis tuberosa* to salinity and UV radiation stresses. *Acta Biologica Szegediensis*, **54**, 37–43.

Hassan IA, Basahi JM, Haiba NS, Kadi MW (2013) Investigation of climate changes on metabolic response of plants: Interactive effects of drought stress and excess UV-B. *Journal of Earth Science & Climatic Change*, **4**, 129.

Hassan IA, Aou-Zeid HM, Basahi JM (2011) Photosynthetic response of Egyptian cultivar of broad bean (*Vicia faba* L.) to UV-B and drought, singly and in combination. *International Research Journal of Agricultural Science and Soil Science*, **1**, 455–461.

He ZS, Zhan SF, Wu SL (2014) Interactive effects of increased UV-B and rainfall on the growth and physiology of Tibetan barley on the Tibetan plateau. *Ekoloji Dergisi*, **23**, 18–26.

Januskaitiene I (2011) Effects of substrate acidity and UV-B radiation on photosynthesis of radishes. *Central European Journal of Biology*, **6**, 624–631.

Kataria S, Guruprasad KN, Ahuja S, Singh B (2013) Enhancement of growth, photosynthetic performance and yield by exclusion of ambient UV components in C3 and C4 plants. *Journal of Photochemistry and Photobiology B: Biology*, **127**, 140–152.

Keiller DR, Holmes MG (2001) Effects of long-term exposure to elevated UV-B radiation on the photosynthetic performance of five broad-leaved tree species. *Photosynthesis Research*, **67**, 229–240.

Kumari R, Singh S, Agrawal SB (2009) Effects of supplemental ultraviolet-B radiation on growth and physiology of *Acorus calamus* L.(sweet flag). *Acta Biologica Cracoviensia Series Botanica*, **51**, 19–27.

Lake JA, Field KJ, Davey MP, Beerling DJ, Lomax BH (2009) Metabolomic and physiological responses reveal multi‐phasic acclimation of *Arabidopsis thaliana* to chronic UV radiation. *Plant, Cell & Environment*, **32**, 1377–1389.

Li X, Zhang L, Li Y, Ma L, Bu N, Ma C (2012) Changes in photosynthesis, antioxidant enzymes and lipid peroxidation in soybean seedlings exposed to UV-B radiation and/or Cd. *Plant and Soil*, **352**, 377–387.

Lidon FC, Ramalho JC (2011) Impact of UV-B irradiation on photosynthetic performance and chloroplast membrane components in *Oryza sativa* L. *Journal of Photochemistry and Photobiology B: Biology*, **104**, 457–466.

Liu Q, Yao X, Zhao C, Cheng X (2011) Effects of enhanced UV-B radiation on growth and photosynthetic responses of four species of seedlings in subalpine forests of the eastern Tibet plateau. *Environmental and Experimental Botany*, **74**, 151–156.

Liu S, Yu L, Liu L, Yang A, Huang X, Zhu A, Zhou H (2023) Effects of ultraviolet-B radiation on the regulation of ascorbic acid accumulation and metabolism in lettuce. *Horticulturae*, **9**, 200.

Lou YS, Wu L, Lixuan R, Meng Y, Shidi Z, Huaiwei Z, Yiwei Z (2016) Effects of silicon application on diurnal variations of physiological properties of rice leaves of plants at the heading stage under elevated UV-B radiation. *International Journal of Biometeorology*, **60**, 311–318.

Lu Y, Duan B, Li C (2007). Physiological responses to drought and enhanced UV-B radiation in two contrasting *Picea asperata* populations. *Canadian Journal of Forest Research*, **37**, 1253–1262.

Lu Y, Duan B, Zhang X, Korpelainen H, Berninger F, Li C (2009) Intraspecific variation in drought response of *Populus cathayana* grown under ambient and enhanced UV-B radiation. *Annals of Forest Science*, **66**, 613.

Mark U, Tevini M (1997) Effects of solar ultraviolet-B radiation, temperature and CO_2_ on growth and physiology of sunflower and maize seedlings. *Plant Ecology*, **128**, 225–234.

Martínez-Lüscher J, Morales F, Delrot S, Sánchez-Díaz M, Gomès E, Aguirreolea J, Pascual I (2013) Short-and long-term physiological responses of grapevine leaves to UV-B radiation. *Plant Science*, **213**, 114–122.

Martínez-Lüscher J, Morales F, Delrot S, Sánchez-Díaz M, Gomès E, Aguirreolea J, Pascual I (2015) Characterization of the adaptive response of grapevine (cv. Tempranillo) to UV-B radiation under water deficit conditions. *Plant Science*, **232**, 13–22.

Musil CF, Kgope BS, Chimphango SBM, Dakora FD (2003) Nitrate additions enhance the photosynthetic sensitivity of a nodulated South African Mediterranean-climate legume (*Podalyria calyptrata*) to elevated UV-B. *Environmental and Experimental Botany*, **50**, 197–210.

Naidu SL, Sullivan JH, Teramura AH, DeLucia EH (1993) The effects of ultraviolet-B radiation on photosynthesis of different aged needles in field-grown loblolly pine. *Tree Physiology*, **12**, 151–162.

Ni Y, Xia R, Li J (2014) Changes of epicuticular wax induced by enhanced UV-B radiation impact on gas exchange in *Brassica napus*. *Acta Physiologia Plantarum*, **36**, 2481–2490.

Ormrod DP, Schmidt AM, Livingston NJ (1997) Effect of UV‐B radiation on the shoot dry matter production and stable carbon isotope composition of two *Arabidopsis thaliana* genotypes. *Physiologia Plantarum*, **101**, 497–502.

Poulson ME, Boeger MRT, Donahue RA (2006) Response of photosynthesis to high light and drought for *Arabidopsis thaliana* grown under a UV-B enhanced light regime. *Photosynthesis Research*, **90**, 79–90.

Qaderi MM, Reid DM (2005) Growth and physiological responses of canola (*Brassica napus*) to UV‐B and CO_2_ under controlled environment conditions. *Physiologia Plantarum*, **125**, 247–259.

Qaderi MM, Basraon NK, Chinnappa CC, Reid DM (2010) Combined effects of temperature, ultraviolet-B radiation, and watering regime on growth and physiological processes in canola (*Brassica napus*) seedlings. *International Journal of Plant Sciences*, **171**, 466–481.

Qaderi MM, Reid DM, Yeung EC (2007) Morphological and physiological responses of canola (Brassica napus) siliquas and seeds to UVB and CO_2_ under controlled environment conditions. *Environmental and Experimental Botany*, **60**, 428–437.

Ranjbarfordoei A, Samson R, Van Damme P (2011) Photosynthesis performance in sweet almond [*Prunus dulcis* (Mill) D. Webb] exposed to supplemental UV-B radiation. *Photosynthetica*, **49**, 107–111.

Ren J, Dai W, Xuan Z, Yao Y, Korpelainen H, Li C (2007) The effect of drought and enhanced UV-B radiation on the growth and physiological traits of two contrasting poplar species. *Forest Ecology and Management*, **239**, 112–119.

Ren J, Duan B, Zhang X, Korpelainen H, Li C (2010) Differences in growth and physiological traits of two poplars originating from different altitudes as affected by UV‐B radiation and nutrient availability. *Physiologia Plantarum*, **138**, 278–288.

Robson TM, Hartikainen SM, Aphalo PJ (2015) How does solar ultraviolet‐B radiation improve drought tolerance of silver birch (*Betula pendula* Roth.) seedlings? *Plant, Cell & Environment*, **38**, 953–967.

Schmidt AM, Ormrod DP, Livingston NJ, Misra S (2000) The interaction of ultraviolet-B radiation and water deficit in two *Arabidopsis thaliana* genotypes. *Annals of Botany*, **85**, 571–575.

Schumaker MA, Bassman JH, Robberecht R, Radamaker GK (1997) Growth, leaf anatomy, and physiology of *Populus* clones in response to solar ultraviolet-B radiation. *Tree Physiology*, **17**, 617–626.

Shen X, Dong Z, Chen Y (2015) Drought and UV-B radiation effect on photosynthesis and antioxidant parameters in soybean and maize. *Acta Physiologiae Plantarum*, **37**, 25.

Shen X, Zhou Y, Duan L, Li Z, Eneji AE, Li J (2010). Silicon effects on photosynthesis and antioxidant parameters of soybean seedlings under drought and ultraviolet-B radiation. *Journal of Plant Physiology*, **167**, 1248–1252.

Skórska E, Grzeszczuk M, Barańska M, Wójcik-Stopczyńska B (2019) Long wave UV-B radiation and Asahi SL modify flavonoid content and radical scavenging activity of *Zea mays* var. *Saccharata* leaves. *Acta Biologica Cracoviensia series Botanica*, **61**, 87–92.

Sullivan JH, Teramura AH (1990) Field study of the interaction between solar ultraviolet-B radiation and drought on photosynthesis and growth in soybean. *Plant Physiology*, **92**, 141–146.

Sullivan JH, Gitz DC, Peek MS, McElrone AJ (2003) Response of three eastern tree species to supplemental UV-B radiation: leaf chemistry and gas exchange. *Agricultural and Forest Meteorology*, **120**, 219–228.

Takshak S, Agrawal SB (2017) Effect of supplemental ultraviolet-B radiation on *Withania somnifera* L.(Dunal): An appraisal of morphological, physiological and biochemical characteristics. *Indian Journal of Experimental Biology*, **55**, 845–852.

Teramura AH, Sullivan JH, Ziska LH (1990) Interaction of elevated ultraviolet-B radiation and CO_2_ on productivity and photosynthetic characteristics in wheat, rice, and soybean. *Plant Physiology*, **94**, 470–475.

Tosserams M, Rozema J (1995) Effects of ultraviolet-B radiation (UV-B) on growth and physiology of the dune grassland species *Calamagrostis epigeios*. *Environmental Pollution*, **89**, 209–214.

Wang S, Zhang S, Liu X, Bai Lu, Qin Q (2024) Effect of UV-B radiation on photosynthesis based flavonoid synthesis in daylily leaves. *Phytochemistry Letters*, **62**, 24–33.

Wang H, Guo Y, Zhu J, Yue K, Zhou K (2021) Characteristics of mango leaf photosynthetic inhibition by enhanced UV-B radiation. *Horticulturae*, **7**, 557.

Wijewardana C, Henry WB, Gao W, Reddy KR (2016) Interactive effects on CO_2_, drought, and ultraviolet-B radiation on maize growth and development. *Journal of Photochemistry and Photobiology B: Biology*, **160**, 198–209.

Xu X, Zhao H, Zhang X, Hänninen H, Korpelainen H, Li C (2010) Different growth sensitivity to enhanced UV-B radiation between male and female *Populus cathayana*. *Tree Physiology*, **30**, 1489–1498.

Yan F, Liu Y, Sheng H, Wang Y, Kang H, Zeng J (2016) Salicylic acid and nitric oxide increase photosynthesis and antioxidant defense in wheat under UV-B stress. *Biologia Plantarum*, **60**, 686–694.

Yao X, Liu Q (2009) Photosynthetic and physiological responses of Swida hemsleyi (C.K. Schneid. et Wangerin) Soják (Cornaceae) subjected to enhanced UV-B radiation and to nitrogen supply. *Polish Journal of Ecology*, **57**, 483–494.

Yao X, Liu Q, Han C (2008) Growth and photosynthetic responses of *Picea asperata* seedlings to enhanced ultraviolet-B and to nitrogen supply. *Brazilian Journal of Plant Physiology*, **20**, 11–18.

Zhao H, Zhao Z, An L, Chen T, Wang X, Feng H (2009) The effects of enhanced ultraviolet-B radiation and soil drought on water use efficiency of spring wheat. *Journal of Photochemistry and Photobiology B: Biology*, **94**, 54–58.
