# Supplementary material for: A Global Meta‐Analysis of Water Use Efficiency Proxies Reveals That UV Radiation Decreases Transpiration Without Improving WUE
Source: Plant Cell Environ. 2025 May 28;48(9):6734–47. doi: 10.1111/pce.15643 (PMC12319297; doi:10.1111/pce.15643)
Supplement: Supplementary file 3 — SuppMat. [file PCE-48-6734-s002.docx]

**Figure 1:**


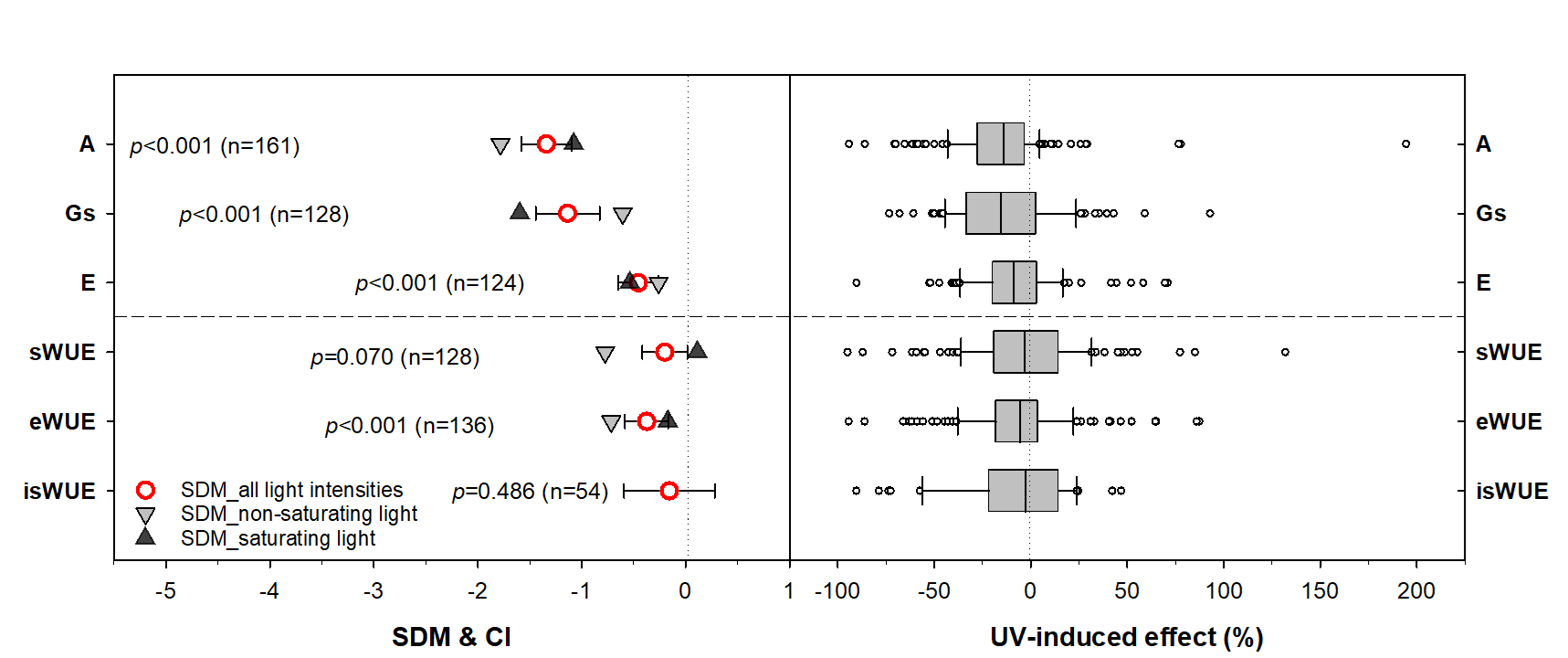


**Figure 1:** The overview of UV effects on CO_2_ assimilation rate (*A*), stomatal conductance (*G*s), transpiration rate (*E*), and water use efficiency calculated on the basis of *G*s (sWUE; *A*/*G*s), *E* (eWUE; *A*/*E*), and carbon stable isotope ratio (isWUE). Left panel: Meta‐analysis significance of UV‐induced effects: SDM, standard difference in means; 95% CI, 95% confidence interval. The numbers indicate the number of case studies included in the meta‐analysis. Right panel: Quantitative changes induced by UV radiation: vertical bars = medians, boxes = inter-quartile range (IQR), whiskers = 5th and 95th percentiles, points = outliers identified.

**Figure 2:**


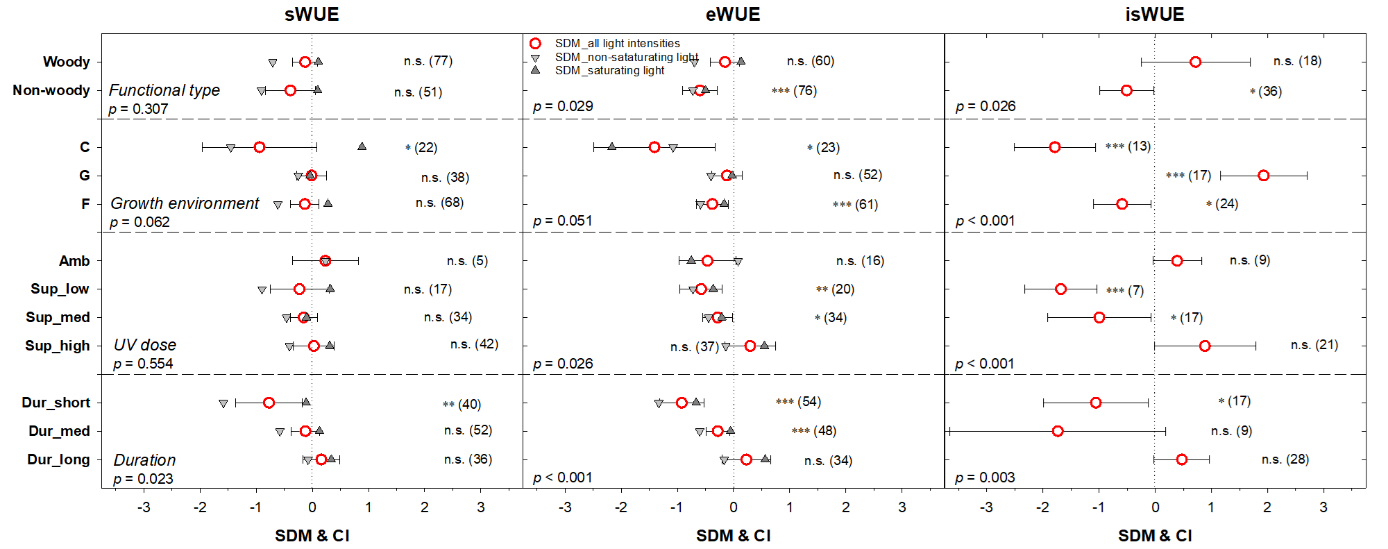


**Figure 2:** A detailed analysis of UV effects on water use efficiency based on stomatal conductance (sWUE), transpiration rate (eWUE), and abundance of stable carbon isotopes (isWUE). Symbols represent standard difference in means (SDMs), with error bars indicating 95% confidence intervals (CIs). Data are categorized by plant functional groups (Woody vs. Non-woody), growth environment (C – growth chamber, G – greenhouse, F – field conditions), applied biologically effective UV dose in kJ m^–2^ day^–1^ (Amb – ambient UV intensity; supplementary doses: Sup_low – below 5 kJ m^–2^ day^–1^; Sup_med – 5–10 kJ m^–2^ day^–1^; Sup_high – above 10 kJ m^–2^ day^–1^, and UV treatment duration (Dur_short ≤30 days; Dur_med 30–90 days; Dur_long >90 days). Asterisks denote statistical significance of UV effects within each group: n.s. – *p* > 0.05, * – 0.05 ≥ *p* > 0.01; ** – *p* ≤ 0.01; *** – *p* ≤ 0.001. Numbers in brackets indicate the count of case studies analysed per group. In addition, numerical *p*-values indicate statistically significant differences within tested categories. For detailed analysis of UV effects on CO_2_ assimilation rate (*A*), stomatal conductance (*G*s), and transpiration rate (*E*), see Figure 4.

**Figure 3:**


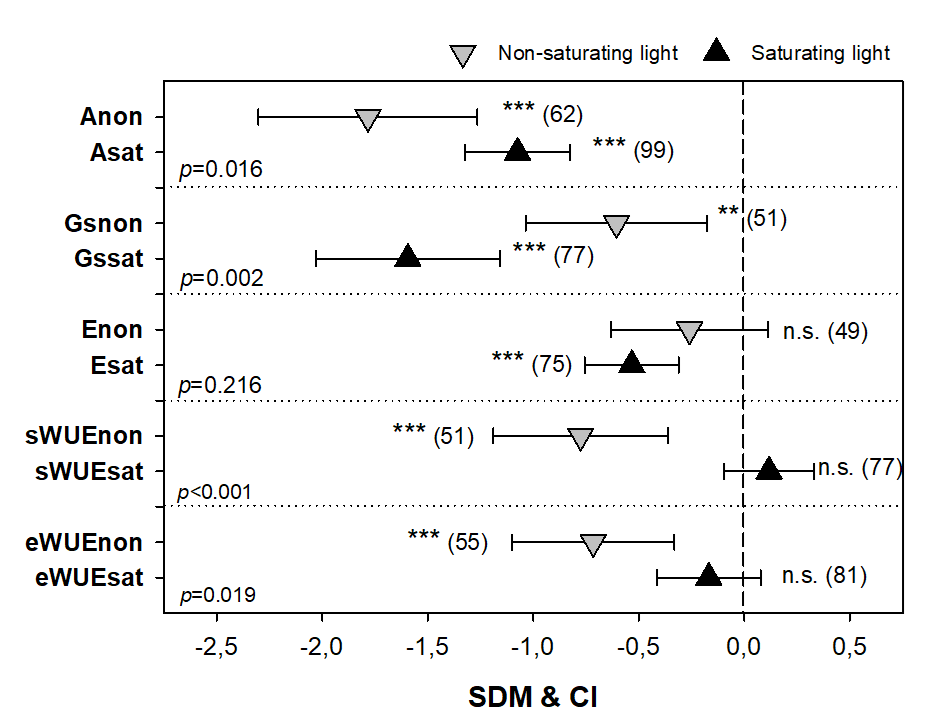


**Figure 3:** Meta‐analysis of UV‐induced effects on CO_2_ assimilation rate (*A*), stomatal conductance (*G*s), transpiration rate (*E*), stomata-based water use efficiency (sWUE; *A*/*G*s), and transpiration-based water use efficiency (eWUE; *A*/*E*). Symbols represent standard differences in means (SDMs) for non-saturating light conditions (light grey triangles; non) and saturating light conditions (dark grey triangles; sat), with error bars showing 95% confidence intervals (CIs). Asterisks indicate the statistical significance of UV effects within each group: n.s. – *p* > 0.05, * – 0.05 ≥ *p* > 0.01; ** – *p* ≤ 0.01; *** – *p* ≤ 0.001. Numbers in brackets show the number of case studies analysed per group. *p*-values on the left indicate statistically significant differences between SDM values under saturating (>1000 µmol m^–2^ s^–1^) and non-saturating intensities of photosynthetically active radiation (PAR).

**Figure 4:**


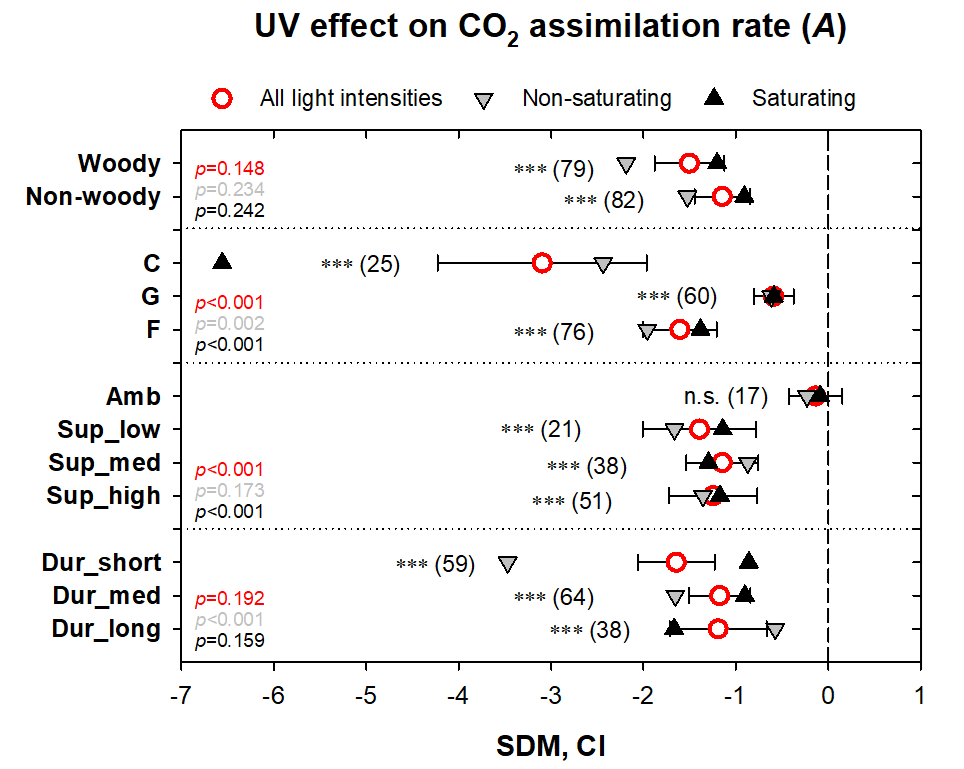


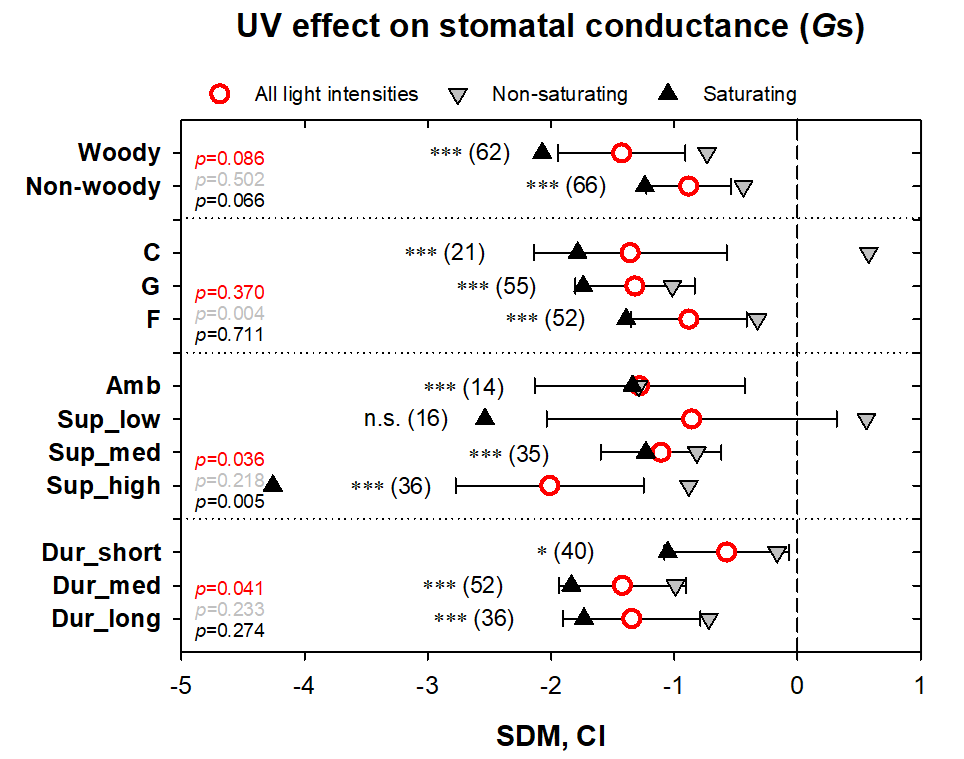


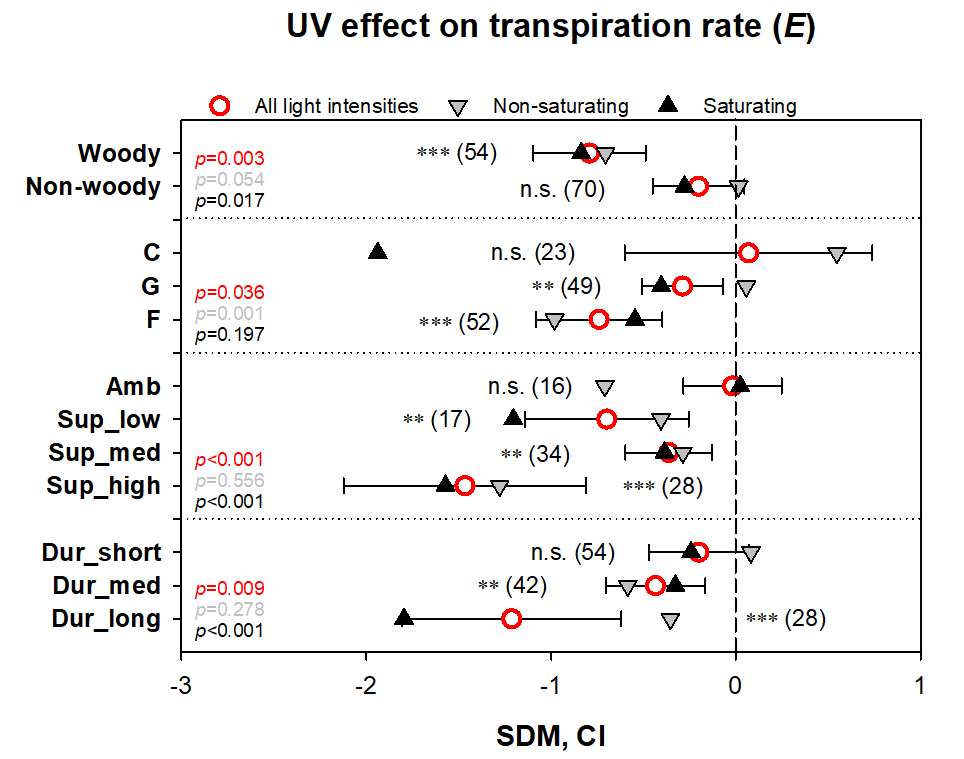


**Figure 4:** Analysis of instantaneous UV effects on CO_2_ assimilation rate (*A*; upper panel), stomatal conductance (*G*s; middle panel), and transpiration rate (*E*; bottom panel) determined via gas-exchange techniques. Symbols represent the standard difference in means (SDMs), with error bars showing 95% confidence intervals (CIs). Red circles indicate SDM values for all studies regardless of light intensity, while light grey and dark grey triangles represent SDM values for studies conducted under non-saturating and saturating light intensities, respectively. All parameters are further categorized by plant functional groups (Woody vs. Non-woody), growth environment (C – growth chamber, G – greenhouse, F – field conditions), applied biologically effective UV dose in kJ m^–2^ day^–1^ (Amb – ambient UV intensity (exclusion type of UV experiments), supplementary doses (Sup_low – below 5 kJ m^–2^ day^–1^; Sup_med – 5–10 kJ m^–2^ day^–1^; Sup_high – above 10 kJ m^–2^ day^–1^), and UV treatment duration (Dur_short ≤30 days, Dur_med 30–90 days, Dur_long >90 days). Asterisks denote the statistical significance of UV effects within each group: n.s. – *p* > 0.05, * – 0.05 ≥ *p* > 0.01; ** – *p* ≤ 0.01; *** – *p* ≤ 0.001. Numbers in brackets indicate the number of case studies analysed per group. Additionally, *p*-values highlight statistically significant UV effects within tested categories for all light conditions (red), non-saturating light conditions (light grey), and saturating light intensities (dark grey).

**Figure 5:**


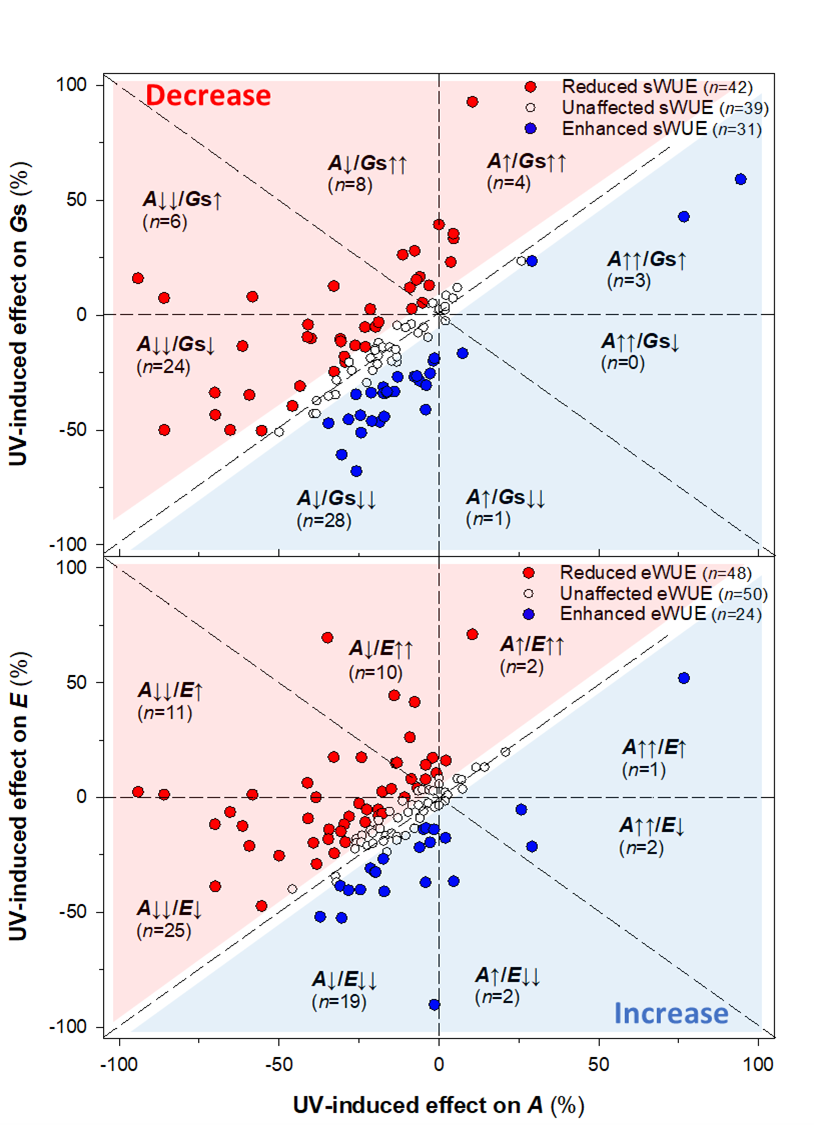


**Figure 5:** Relationship between UV-induced effects on CO_2_ assimilation rate (*A*) and stomatal conductance (*G*s; upper panel) or transpiration rate (*E*; lower panel). White circles indicate case studies where UV effects on water use efficiency (WUE) ranged between -10% (decrease) and +10% (increase), reflecting proportional changes in *A* and *G*s (sWUE) or *A* and *E* (eWUE). Red (blue) circles represent case studies showing a UV-induced decrease (increase) in WUE due to disproportional changes in *A*/*G*s or *A*/*E*. Arrows depict increase (↑) or decrease (↓) in each parameter within all segments of the graph, with double arrows indicating stronger UV effects. The total number of case studies per category is indicated by *n*-values.

**Supplementary Figure S1:**


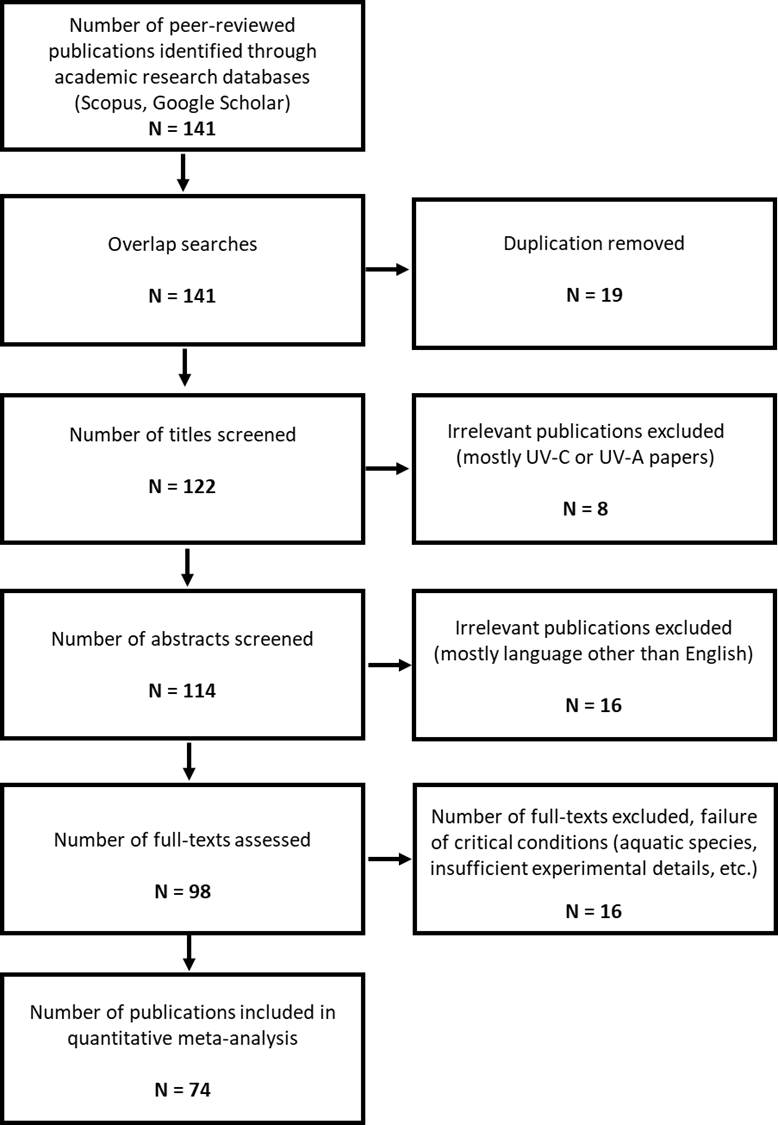


**Supplementary Figure S1:** PRISMA flow diagram illustrating the literature screening process across various phases of the analysis. The screening resulted in the inclusion of 74 publications in the meta-analysis of UV effect on water use efficiency in terrestrial plants. Additional details regarding the selection criteria and process are provided in the main text.

**Supplementary Figure S2:**

**
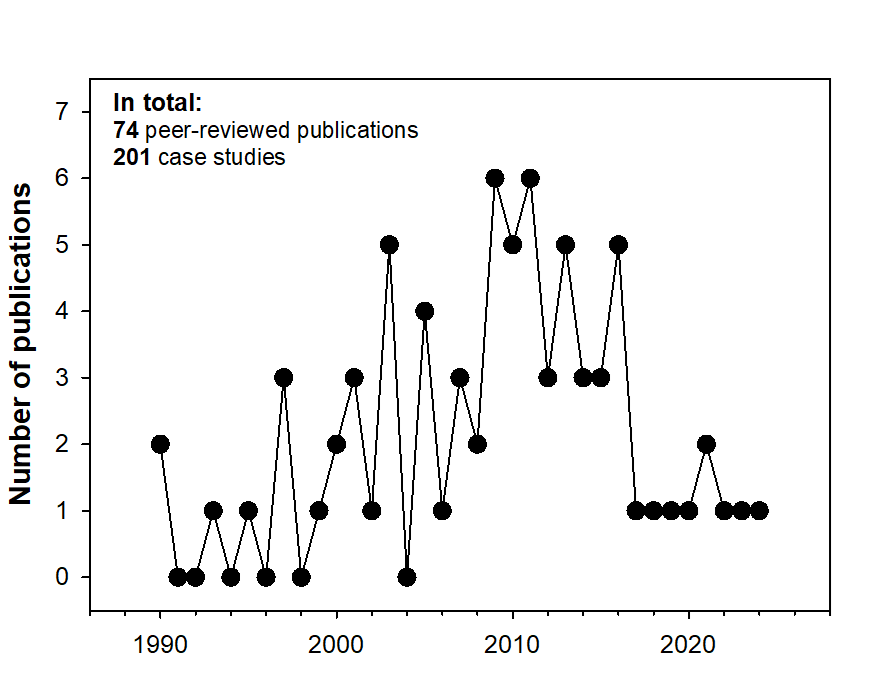
**

**Supplemental Figure S2:** A total of 74 research papers published in peer-reviewed journals between 1990 and 2024 were included in the meta-analysis. Studies reporting on multiple plant species/cultivars, UV treatment durations, or growth condition were treated as separate ‘case studies’, resulting in 201 case studies. Each case study provided data on at least one type of water use efficiency: stomata-based (sWUE), transpiration-based (eWUE), or isotope-based (δ^13^C; isWUE). The dataset includes 127 case studies on sWUE, 137 on eWUE, and 54 on isWUE. Additionally, sWUE and eWUE parameters were categorized according to the instantaneous light intensity conditions during gas-exchange measurements, categorized as saturating (sat) and non-saturating (non) light intensities: sWUEnon – 50 studies, sWUEsat – 77 studies, eWUEnon – 56 studies, eWUEsat – 81 studies.

**Supplementary Figure S3:**

**
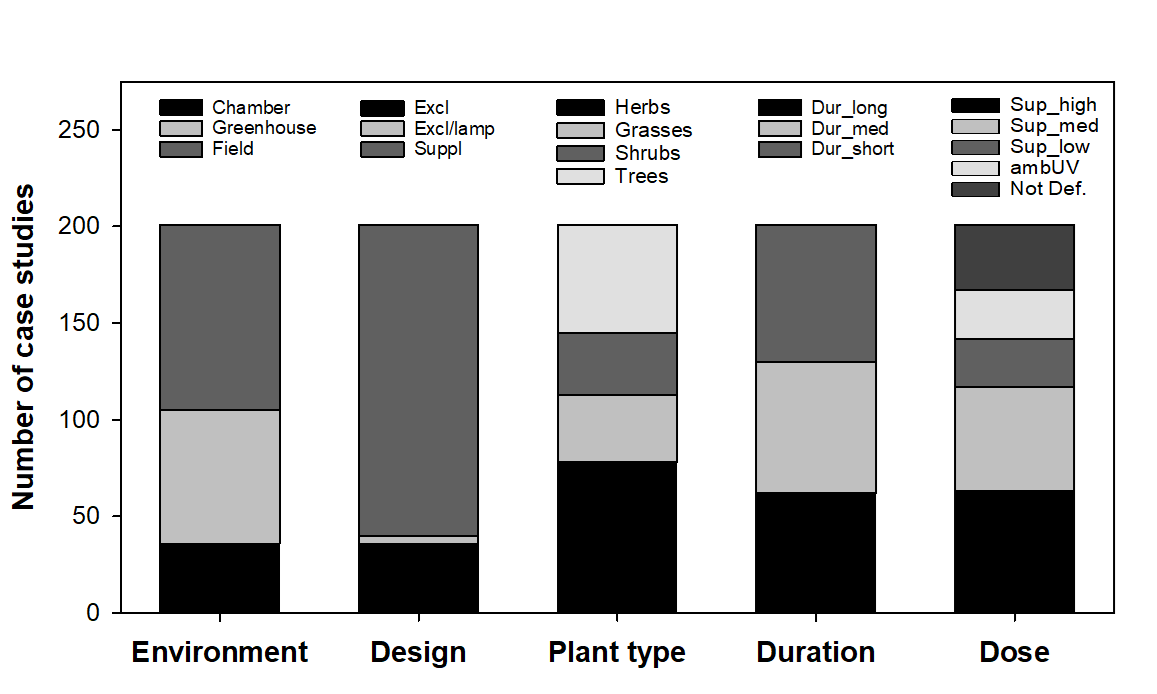
**

**Supplemental Figure S3:** Overview of case studies included in the meta-analysis. A total of 36, 69, and 96 case studies were conducted under growth-chamber, greenhouse, and open-field conditions, respectively. The majority of studies (161) used supplementary filtered UV lamps to enhance UV radiation (Suppl), while 36 studies excluded UV radiation (Excl) by filtering ambient UV. Four case studies combined Excl design with an auxiliary UV light source. Model/experimental plant species included herbs (78), grasses (35), shrubs (32), and trees (43 broadleaved and 13 coniferous species). The database comprises 62 long-term (>90 days; Dur_long), 68 mid-term (30–90 days; Dur_med), and 71 short-term (≤30 days; Dur_short) case studies. In 34 studies, the biologically effective UV dose in kJm^–2^ day^–1^ was not reported (Not def.). Supplemental UV doses were categorized as Sup_low (below 5 kJ m^–2^ day^–1^; 25 studies), Sup_med (5–10 kJ m^–2^ day^–1^; 54 studies), or Sup_high (above 10 kJ m^–2^ day^–1^; 63 studies), while ambient UV doses are referred to as ambUV.

**Supplementary Figure S4:**


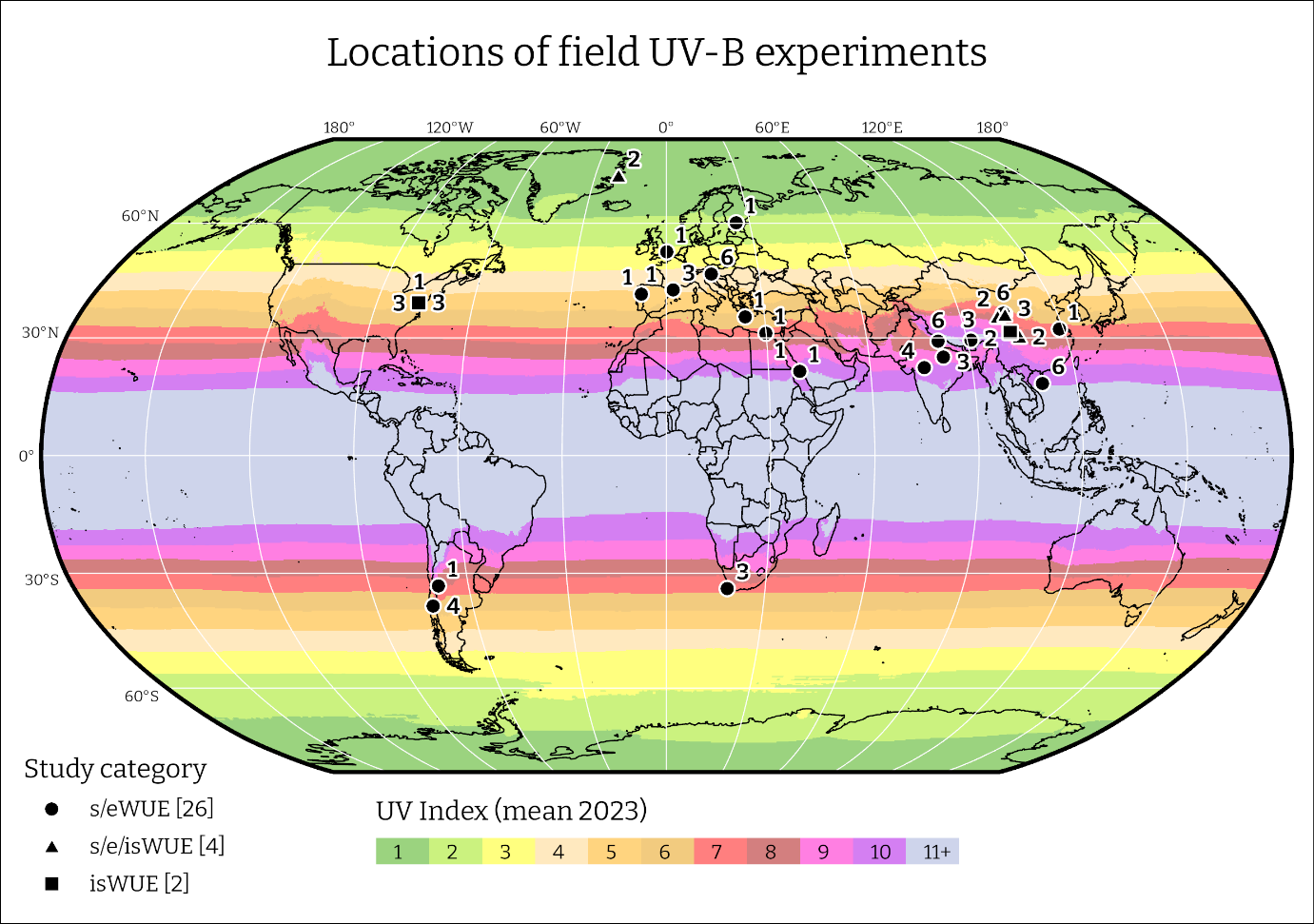


**Supplementary Figure S4:** Global locations of UV-B field experiments included in the meta-analysis. Black circles indicate 26 studies that calculated stomatal (sWUE) and transpirational water use efficiency (eWUE), black triangles represent 4 studies that calculated stomatal, transpirational, and isotopic WUE, while squares denote 2 studies that calculated isotopic WUE. The colour scale represents the average UV index for 2023. (Source: KNMI/TEMIS)

**Supplementary Figure S5:**


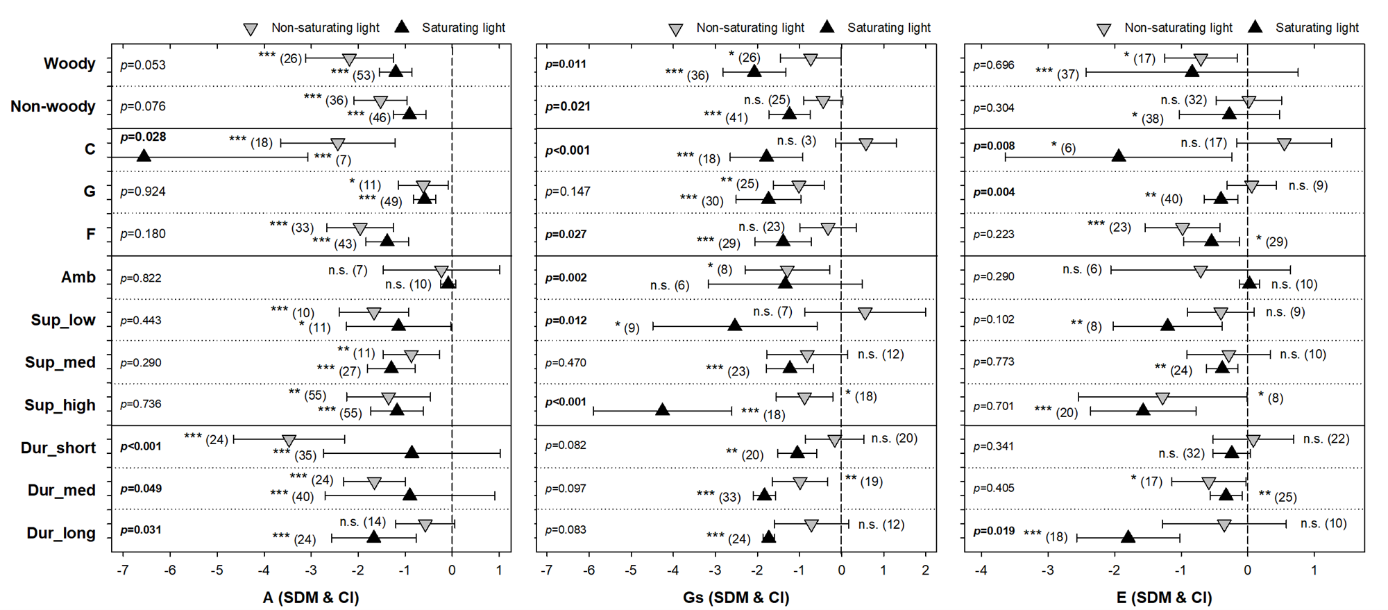


**Supplementary Figure S5:** Analysis of the effects of photosynthetically active radiation intensity during gas-exchange measurements of CO_2_ assimilation rate (*A*; left panel), stomatal conductance (*G*s; middle panel), and transpiration rate (*E*; right panel) in UV-treated plants. Light grey and dark grey triangles represent SDM (Standardized Mean Difference) values for studies conducted under non-saturating and saturating light intensities, respectively, with error bars indicating 95% confidence intervals (CIs). *p*–values indicate statistical significance of differences between non-saturating and saturating light intensities for each category, including plant functional type, growth environment, applied biologically effective UV dose, and UV treatment duration. For detailed descriptions of these categories, refer to Figure 2. Statistically significant differences (*p* < 0.05) are highlighted in bold. Asterisks denote the significance of the UV effect (n.s. – *p* > 0.05, * – 0.05 ≥ *p* > 0.01; ** – *p* ≤ 0.01; *** – *p* ≤ 0.001). Numbers in brackets indicate the total number of case studies included.

**Additional supplementary materials – available online**

**Supplementary Table S1:** Dataset for the paper by Jansen et al.: A global meta-analysis of Water Use Efficiency proxies reveals that UV radiation decreases transpiration without improving WUE

**Reference list** to Supplementary Table S1
